# TOX4 and NOVA1 Proteins Are Partners of the LEDGF PWWP Domain and Affect HIV-1 Replication

**DOI:** 10.1371/journal.pone.0081217

**Published:** 2013-11-27

**Authors:** Mehdi Morchikh, Monica Naughtin, Francesca Di Nunzio, Johan Xavier, Pierre Charneau, Yves Jacob, Marc Lavigne

**Affiliations:** 1 Ecole Normale Supérieure, Laboratoire Joliot-Curie, Centre National de la Recherche Scientifique, Lyon, France; 2 Institut Pasteur, Unité de Virologie Structurale, Centre National de la Recherche Scientifique, Unité de recherche associée, Paris, France; 3 Université Pierre et Marie Curie, Paris, France; 4 Institut Pasteur, Unité de Virologie Moléculaire et Vaccinologie, Centre National de la Recherche Scientifique, Paris, France; 5 Institut Pasteur, Unité de Génétique Moléculaire des Virus à ARN, Centre National de la Recherche Scientifique, Paris, France; 6 Center for Cancer Systems Biology and Department of Cancer Biology, Dana-Farber Cancer Institute, Boston, Massachusetts, United States of America; Centro de Biología Molecular Severo Ochoa (CSIC-UAM), Spain

## Abstract

PWWP domains are involved in the chromatin attachment of several proteins. They bind to both DNA and proteins and their interaction with specific histone methylation marks define them as a new class of histone code readers. The lens epithelium derived growth factor (LEDGF/p75) contains an N-terminal PWWP domain necessary for its interaction with chromatin but also a C-terminal domain which interacts with several proteins, such as lentiviral integrases. These two domains confer a chromatin-tethering function to LEDGF/p75 and in the case of lentiviral integrases, this tethering participates in the efficiency and site selectivity of integration. Although proteins interacting with LEDGF/p75 C-terminal domain have been extensively studied, no data exist about partners of its PWWP domain regulating its interaction with chromatin. In this study, we report the identification by yeast-two-hybrid of thirteen potential partners of the LEDGF PWWP domain. Five of these interactions were confirmed in mammalian cells, using both a protein complementation assay and co-immunoprecipitation approaches. Three of these partners interact with full length LEDGF/p75, they are specific for PWWP domains of the HDGF family and they require PWWP amino acids essential for the interaction with chromatin. Among them, the transcription activator TOX4 and the splicing cofactor NOVA1 were selected for a more extensive study. These two proteins or their PWWP interacting regions (PIR) colocalize with LEDGF/p75 in Hela cells and interact *in vitro* in the presence of DNA. Finally, single round VSV-G pseudotyped HIV-1 but not MLV infection is inhibited in cells overexpressing these two PIRs. The observed inhibition of infection can be attributed to a defect in the integration step. Our data suggest that a regulation of LEDGF interaction with chromatin by cellular partners of its PWWP domain could be involved in several processes linked to LEDGF tethering properties, such as lentiviral integration, DNA repair or transcriptional regulation.

## Introduction

The PWWP domain is a 70–135 amino acid sequence containing the Pro-Trp-Trp-Pro (PWWP) motif, which is conserved between more than 60 eukaryotic proteins characterized for their DNA or chromatin interaction [1]. Initially discovered in the HDGF and WHSC1 proteins [2], [3], it has been recently characterized as a new “reader” of specific histone methylations [1], [4]–[9]. Several studies have highlighted the role of this domain in different nuclear processes, such as DNA methylation or repair, transcription regulation or retroviral integration.

Crystal and NMR structures of this domain have revealed a structural organization in two parts, an N-terminal five stranded beta-barrel core and a C-terminal alpha-helix bundle. The PWWP domains can be categorized into six classes based on sequence homology [1] that mainly differ by a peptidic sequence inserted between the 2^nd^ and 3^rd^ beta strands of the beta-barrel or by their location within the protein.

PWWP domains interact with both DNA and proteins. DNA interaction was originally shown for the DNMT3b PWWP domain [10], [11] and recent structural studies of several PWWP domains have revealed the presence of a positively charged surface enriched in basic residues and involved in this DNA binding property [12]–[16]. PWWP domains were initially proposed to be responsible for protein-protein interaction [17]. This hypothesis was confirmed by the identification of several PWWP protein partners, such as SAL3 [18], the SUMOE3 ligase PIAS1 [19] and canonical histones [20]. Genetic, biochemical and structural studies of these interactions have shown the role of an aromatic cage formed by conserved hydrophobic residues [1], [7], [14], [15]. Recently, PWWP domains have been characterized as new histone code readers. They recognize specifically methylated histones, a property conserved with other members of the Tudor domain “Royal family” such as the Chromo, MBT or tudor domains [21], [22]. For example, the PWWP domains of DNMT3A, BRPF1, IOC4 or LEDGF proteins specifically interact with Histone 3 trimethylated on Lysine 36 (H3K36me3) [1], [4]–[9] and PDP1 PWWP domain binds to Histone 4 trimethylated on Lysine 20 (H4K20me3) [14]. Histone-PWWP affinities are weak but the PWWP-nucleosome complexes are stabilized by additional PWWP-DNA interactions, as shown with the LEDGF PWWP domain [8], [9].

In addition to the PWWP domain, the N-terminal part of LEDGF contains other DNA binding domains, including two AT hooks and three positively charged regions (CR) that contribute to its tight chromatin association throughout the cell cycle [23], [24]. There are two isoforms of LEDGF, p52 and p75 that share this N-terminal chromatin and DNA binding part, but differ by their C-terminus. Both forms were discovered as transcription co-activators but they interact with different proteins. The shortest form of LEDGF, p52, binds to the splicing factor SRSF1 and several other proteins involved in mRNA processing [6], [25]. The longest form, p75, interacts with several cellular proteins involved in transcriptional regulation (JPO2 or Menin-MLL complex), DNA replication (Cdc7-Activator of S-phase Kinase ASK), and DNA transposition (PogZ) [26]-[29] but also with lentiviral integrases [30], [31]. These interactions occur through the C-terminal part of LEDGF, called Integrase Binding Domain (IBD) [32]. In the case of HIV-1, LEDGF/p75 plays a key role during its replication [33]–[36] and is involved in the choice of integration sites [36]–[39]. LEDGF functions as a bimodal protein, interacting with lentiviral integrases (via the IBD) and tethering them to cellular chromatin (via the PWWP). While the Integrase-IBD interaction has been extensively studied and has been described as a new antiviral target [40]–[45], the PWWP-chromatin interaction is still under investigation. The recent identification of a specific recognition by this domain of the H3K36me3 mark has challenged the previous model involving an additional cellular protein partner [46]. However, both models are compatible and this partner could play a regulatory role, as suggested by studies showing the complex network between readers and writers of histone modifications [47].

To address this question, we looked for cellular partners of the LEDGF PWWP domain. Among the thirteen peptidic sequences identified by yeast two-hybrid approach, five interacted with the LEDGF PWWP domain *in vivo* in 293T cells and three of them kept this interaction with full length LEDGF/p75 protein, were specific for the PWWP domains of HDGF family and lost interaction with PWWP domains mutated in chromatin-binding surface. Due to their role in the regulation of gene expression we focused our study on the TOX4 and NOVA1 proteins and compared their cellular localization and chromatin attachment with LEDGF/p75. Overexpression of the corresponding PWWP Interaction Region (PIR)s inhibited single round VSV-G pseudotyped HIV infection and suggested a physiological role of these proteins as regulators of the LEDGF-chromatin interaction.

## Materials and Methods

### Ethics statment

Healthy peripheral blood was obtained from the Etablishement du Sang Français (EFS, Lyon France) after obtaining patients' written informed consent in accordance with the Declaration of Helsinki.

### Yeast two-hybrid screening

WT and mutated (K14AK16A, W21A, I42AF43A and A51P) LEDGF PWWP domains (aa1 to 100) were cloned by recombinatorial cloning (Gateway^™^ system, Life technology) protocol into the pGBKT7 plasmid and were used as baits against a human brain MATCHMAKER^™^ cDNA library (HY4004AH from Clontech) present in a pGADT7 vector. Yeast two-hybrid screening was performed in AH109 yeast strain using a cell-to-cell mating protocol adapted from [48] and the selectivity of HIS3 reporter gene was modulated by the presence of 5 mM 3-aminotriazole (Sigma). From 214 clones obtained on the 5 PWWP sequences, 140 cDNA sequences were recovered by PCR and sequenced and 13 cDNA sequences coding for PWWP Interacting Regions (PIR) were selected for further studies (listed in Table S1).

### Plasmid constructions

- pHA-LEDGF and pGEX-4T-PWWP LEDGF were obtained from A. Engelman (Boston, USA) and have been described previously [15], [49]. LEDGF sequences 1–100 (WT and mutants), 1–176, 1–325 and 1–530 were cloned into the pDonR207 plasmid (by PCR and Gateway^™^ BP reaction), these entry clones were further introduced in different destination vectors. cDNA sequences coding for other PWWP domains used in this study and obtained form different origins were also cloned into the pDonR207 plasmid: aa 1–99 of human HRP2 (A. Engelman; Boston, USA); aa 41–105 of human HDGF (T-H Huang, Taiwan, ROC); aa 41–163 of saccharomyces pombe PDP1 (S. Jia, New-York, USA); aa 86–198 of human MSH6 (B. Gilquin, Saclay, France); aa 211–314 of human NSD2 or WHSC1 (A. Engelman, Boston, USA), aa 214–321 of human DNMT3B (clone Image CH3-b6, Geneservice, Cambridge UK) and aa 233–325 of human BS69 (clone Image AT46-c3, Geneservice, Cambridge UK).

- TOX4 and NOVA1 full-length cDNA sequences were obtained from Image clones (AT17-b8 and BU2-h9 respectively) and were purchased at Geneservice (Cambridge-UK). The thirteen selected PIRs, and TOX4 and NOVA1 full length sequences were cloned into the pDonR207 plasmid (by PCR and Gateway^™^ BP reaction).

- LEDGF constructs (FL, 1–325, 1–116 and PWWP) and the thirteen identified PIRs, present in pDonR207 were transferred respectively into the pSPICA-N1 and pSPICA-N2 acceptor plasmids (by LR Gateway^™^ reaction) allowing to express N-ter Gluc1 or Gluc2 tagged fusion proteins [50].

- TOX4, NOVA1, BC063132, CNRIP1, COP5, NOVA1, RLF and TOX4 PIRs present in pDonR207 were transferred by LR Gateway^™^ into pCiNeo-3Flag allowing to express N-terminal triple-Flag fusions of these proteins in 293T cells. TOX4 and NOVA1, PIR and full length proteins were cloned by restriction/ligation into pET28 acceptor vector allowing their expression in *E. Coli* BL21-DE3 strain.

### Cells and cell culture

293T, Jurkat, Hela and SHSY5Y cells were purchased at ATCC. The P4-CCR5 reporter cells are HeLa CD4+ CXCR4+ CCR5+ carrying the LacZ gene under the control of the HIV-1 long terminal repeat (LTR) promoter [51]. Peripheral Blood Mononuclear Cells (PBMC) were isolated by ficol gradient from healthy peripheral blood, obtained from the Etablishement du Sang Français (EFS, Lyon France) after obtaining patients' written informed consent in accordance with the Declaration of Helsinki.

293T, Hela and Jurkat cells were maintained in a humidified atmosphere at 37°C with 5% CO_2_. Medium used for the growth were Dulbecco's modified minimal essential medium (*Gibco or PAA*) for 293T cells, MEM alpha modified medium (*Gibco or PAA*) for Hela and RPMI (Roswell Park Memorial Institute) 1640 for Jurkat cells, supplemented with 1% L-glutamine (*Gibco or PAA*), 1% penicillin, 1% streptomycin (*Gibco*) and 10% fetal bovine serum (PAA, A10110-2569) for 293T and Jurkat cells plus 1% non-essential amino acids (*PAA*) for Hela cells.

### Lentiviral Vectors Construction and Production

LV vectors are based on the pFLAP_CMV_EGFP_WPRE vector, which is ΔU3, contains the cis-acting sequences required for formation of the central DNA Flap, and encodes the enhanced green fluorescent protein (eGFP) under the control of the CMV promoter to monitor transduction. Transgenes TOX4 PIR, TOX4 HMG, NOVA1 PIR and LEDGF IBD were cloned by restriction/ligation into the pFLAP_CMV_EGFP_WPRE lentiviral vector in order to obtain a stable expression of EGFP N-terminal fusion of these constructs. All sequences were cloned using the restriction sites *Age*I and *Xho*I blunt-end of pFLAP_CMV_EGFP_WPRE vector. LVs were produced by transient transfection of 293T cells with the vector, encapsidation (pCMVΔR 8.74), and VSV-G plasmids. Vectors were harvested 48 hr post-transfection and concentrated by ultracentrigation for 1 hr at 64,000 g (Beckman Coulter) at 4°C. LVs were titered in HeLa P4-CCR5 cells using flow cytometry to assess GFP expression at 4 days post-transduction (p.t).

### Protein Complementation Assays

PCA assay were performed as described [50]. 293T cells lined were seeded at 32,000 cells per well in 96-well plates. After 24 h, cells were transfected by linear PEI (polyethylenimine) with 100ng of pSPICA-N1-LEDGF and 100 ng of pSPICA-N2-cellular protein, for expression of the Gluc1-LEDGF/p75 and GLuc2-fusion proteins, where Gluc1 and Gluc2 are two inactive fragments of the Gaussia princeps luciferase. 24 h post-transfection. Cells were lysed in 30 μL of Renilla luciferase lysis buffer (Promega) for 30 minutes. The Gaussia princeps luciferase activity was measured on 30 μL of total cell lysate by a luminometer Berthold Centro XS LB960 after injection of 100 μL of the Native coelenterazine substrate (Promega, #E2820). Results were expressed as a normalized luminescence ratio (NLR). The NLR represents the average luminescence signal detected in cells transfected with pSPICA-N1-LEDGF and pSPICA-N2-Cellular protein divided by the average of luminescence measured in control wells transfected with pSPICA-N1-LEDGF and an empty pSPICA-N2 vector with those transfected with pSPICA-N2-cellular protein and an empty pSPICA-N1 vector. NLR = (Gluc1-LEDGF+Gluc2-cellular proteins)/[(Gluc1-LEDGF+Gluc2 empty)+(Gluc1 empty + Gluc2-Cellular protein)] as described in [50].

### Cellular fractionation assay

Hela cells were seeded in 6-well plates (1.5×10^5^ cells per well) 24 hours before transfection, then transfected with 2 µg plasmid DNA and 4 µg of Jetprime reagent (Polyplus) per well. Cells were fractionated 24 hr post-transfection using the method firstly described by [23] and modified by [15]. The total protein concentration of each fraction was determined by Bradford assay. 30 µg (for endogenous proteins) or 5µg (for ectopically expressed proteins) of each fraction were analyzed by Western blotting with anti-TOX4 antibody (Sigma HPA017880 1∶1000), anti-NOVA1 antibody (Abcam Ab97368, 1∶700), anti-LEDGF antibody (BD #611714 1∶2000), anti-FLAG M2 antibody (Sigma, A2220 1∶2000) or anti-HA antibody (Sigma, 1∶2000).

### Endogenous Western blot of TOX4 and NOVA1

Lysates from Hela, SHSY5Y brain, and Jurkat cell lines, and stimulated PBMC cells taken from two donors were harvested in radioimmunoprecipitation assay (RIPA) buffer (20 mM Hepes, 150 mM Tris-HCl [pH 8.0], 150 mM NaCl, 1% [wt/vol] deoxycholate, 0.1% [wt/vol] sodium dodecyl sulfate, 1% [vol/vol] NP-40), 2 mM EDTA). Total protein concentration was determined by Bradford assay and 30 µg of protein was Western blotted using either anti-TOX4 antibody (Sigma HPA017880 1∶1000), anti-NOVA1 antibody (Abcam Ab97368, 1∶700) or anti-B-actin antibody (Sigma, 1∶2000).

### Epifluorescence Microscopy

Hela cells were seeded in 24-well dishes on sterilized glass coverslips (8×10^4^ cells per well) 24 hours before transfection, then transfected with 0.5µg total plasmid DNA and 1µg of Jetprime reagent (Polyplus). 24 hours post-transfection cells were fixed with 4% paraformaldehyde for 10 min. Cells were then washed twice in PBS and permeabilized in 0.1% (vol/vol) Triton X-100 in PBS for 10 min. Cells were blocked for 30 minutes at 37°C in blocking buffer containing 10% FCS, 3% BSA, 0.1% (vol/vol) Triton X-100 in PBS. Cells were stained with primary antibodies for 30 minutes at 37°C, followed by washing (3× 10 min in PBS-Triton X-100 0.1%), and detection with secondary antibodies coupled with Alexa dyes (A488, A555 or A647) (Molecular Probe, dilution 1∶2000). Primary antibody dilutions were as follows; anti-TOX4 antibody (Sigma HPA017880 1∶200), anti-NOVA1 antibody (Abcam Ab97368, 1∶200), anti-LEDGF mouse antibody (BD #611714 1∶200), anti-LEDGF rabbit (Bethyl A300-848 1∶200), anti-SC35 (Abcam Ab11826, 1∶200), anti-Coilin (Abcam Ab87913, 1∶200), anti-FLAG M2 antibody (Sigma, A2220 1∶1000), anti-HA rat (Roche clone 3F10, 1∶500). After secondary incubation coverslips were washed again (3×10 min in PBS-Triton X-100 0.1%), rinsed in ddH2O and briefly dipped in 100% ethanol. After drying, coverslips were mounted in Fluromount G medium (Electron Microscopy Science) containing 400 ng/ml DAPI (Sigma). Z-stack images taken at 200 nm steps (pixel size 102 nm) were captured with an Axio-Imager microscope (Zeiss) with a 63× oil objective (NA = 1.4). Images were cropped using Image J (v1.47p ImageJ, US NIH, Bethesda, MD, http://rsb.info.nih.gov/ij/)., then deconvolved using Metamorph version 7.1 software (Meinel algorithm, 7 iterations). Co-localization analysis was perfomed using the JACoP plugin in Image J. Images represented in figures are a maximum z-projection of 5 deconvolved z-stack images surrounding the focal plane.

### Co-immunoprecipitations assays, and western blots

We used 293T cells for protein-protein interaction experiments because protein expression levels were higher than in Hela Cells. 293T cells (4×10^6^) were plated into 15-cm dishes ≥24 h before transfecting 10µg of pHA-LEDGF FL and 10µg of LEDGF partners in pCineo-3XFLag (NOVA1, NOVA1 PIR, TOX4, TOX4 PIR) or pSG-HIV integrase [52] or pFLAG-CMV2-Brd4 [53] using jetPEI (Ozyme). The cells were collected by PBS supplemented with 5 mM EDTA, washed once wash with PBS supplemented with 1 mM PMSF and 1X complete Mini EDTA-free protease inhibitor cocktail (Roche). For the LEDGF-(NOVA1 or TOX4) two 15 cm dishes were lyzed in 600µl of TNEM buffer (50 mM Tris HCl pH8, 0.5 mM EDTA, 0.1% NP-40, 1 mM PMSF) supplemented with 300 mM NaCl and 1X complete Mini EDTA-free protease inhibitor cocktail (Roche) during 30 min with rotation at 4°C. Lysates were then clarified by 15 minutes centrifugation at 16000 g. Lysates were adjusted to 150 mM mM by adding 600µl of TNEM buffer without salt.

60µl of the total extract was kept (5% input) and the total extract was divided into three fractions. The first fraction was incubated during 20 min at 37°C and the two others were treated during 20 min at 37°C with Turbo DNAase (0.17 U/µl, Ambion, AM2238) or RNAse A (10 µg/ml, Sigma). For the co-immunoprecipitation, The total extract was incubated overnight with 10 µl of anti-flag M2-agarose beads (Sigma) with rotation at 4°C and washed 3 times with 1 ml of TNEM supplemented with 150 mM NaCl and boiled in Laemmli buffer plus β-mercaptoethanol. Samples were separated by SDS-polyacrylamide 10% or 7.5% gel electrophoresis and analyzed by Western blotting with anti-Flag M2 (sigma, A2220), anti-HA rat (Roche clone 3F10).

### Protein purification and GST pull-down assays

GST-PWWP was produced in BL21 E. *coli* and purified on Glutathione-Agarose beads and Superdex S200 as described in [15]. Control GST protein was purified on Glutathione-Agarose beads. N-terminal hexa-histidine tagged TOX4 and NOVA1 PIRs were produced in Rosetta *E coli* strain transformed by corresponding pET28 derived plasmids, grown until OD_600nm_ =  0.6 and induced with 0.5 mM IPTG during 3 hours at 30°C. Cells were resuspended in 500 mM NaCl, 25 mM Tris-HCl pH 7.4, 1 mM EDTA, 5 mM DTT, 0.2 mM PMSF, 10 mM Imidazole, 1X protease inhibitor cocktail (Roche), lysed by sonication and centrifuged twice (10 000 g, 30 min). His-tagged proteins were purified from supernatant on 1 ml His-Trap FF crude column (protocol GE Healthcare) with an elution by a linear Imidazole gradient (10-500 mM). Selected fractions were dialyzed against 150 mM NaCl, 25 mM Tris-HCl pH 7.4, 1 mM EDTA, 1 mM DTT and 0.2 mM PMSF and then against the same buffer supplemented with 20% glycerol. An additional size exclusion chromatography was performed on Superdex S200 10/300 GL for TOX4 PIR and Superdex S75 10/300 GL for NOVA1 PIR in the same buffer according to the protocol described by the manufacturer (GE Healthcare). The quality of purified PIRs was checked by SDS-PAGE electrophoresis and either coomassie staining or Western blotting and hybridization with anti-His (Sigma, H1029), anti-TOX4 (Sigma, HPA017880) or anti NOVA1 (Abcam, ab77594) antibody. Polynucleosomes were assembled by salt dialysis on the 2.6 kbp 5SG5E4 DNA using native Hela histones as described in previous studies [49].

GST pull down were performed as described in [32] and adapted in [15]. Interactions between GST proteins and PIRs were tested at 150 mM NaCl, 25 mM Tris-HCl pH 7.4, 5 mM MgCl2, 0.1% NP40, 100µg/ml BSA, 1 mM DTT and 0.2 mM PMSF. Amounts of GST proteins, PIRs or 293T cells extracts containing PIRs, DNA or RNA are described in the corresponding figure's legend. When indicated, 293T extracts were treated during 20 min at 37°C with Turbo DNAse (0.17 U/µl, Ambion, AM2238) or RNAse A (10µg/ml, Sigma), before their incubation with GST proteins attached to the beads.

### Viruses and cell transduction

The viral molecular clones used were based on LAI and called HIV-1 (wild-type), HIV-1-Luc, which contains the luciferase gene at the place of *Nef*, and LAIδenv [54]. Viruses were produced by transient transfection of 293T cells using calcium phosphate precipitation with proviral plasmid alone or co-transfected with the Vesicular Stomatitis Virus glycoprotein (VSV-G) envelope expression plasmid pHCMV-G [55]. Viruses were harvested at 48 hr post-transfection and treated with 25 U/mL of DnaseI (Roche) and 100 mM MgCl2 for 30 min at 37°C. Virus yield was measured by p24 ELISA according to the manufacturer's instructions (Perkin Elmer). Hela P4-CCR5 cells were transfected in presence of lipofectamine 2000 (Invitrogen) with plasmids expressing the following domains, IBD, NOVA1 PIR and TOX4 PIR. Forty-eight hours after transfection 2 million of cells were challenged with 500 ng of p24 antigen of HIV-1-Luc.

Retroviral vector, MLV Luc, derived from Moloney was produced by co-transfection in 293T cells with calcium phosphate of pFBluc 10 mg, pCG gag-pol 10 mg, pMD2 VSV-G.

#### Luciferase assays

Luciferase (Promega) activity was measured 48 hr p.i according to manufacturer's instructions, using a microplate ﬂuorimeter (Victor, Perkin Elmer). Protein quantification by Bio-Rad protein assay was carried out on the same lysates to normalize the luciferase data for protein content.

#### Quantitative PCR

Infected cells and control infected cells cultured in the presence of 5 µM nevirapine were treated for 30 min at 37°C with 1000 U of DnaseI (Roche). Total cellular DNA was then isolated using the QIAamp DNA micro kit (QIAGEN). Two long terminal repeat (2-LTR) containing circles were detected using primers MH535/536 and probe MH603 [56], using as standard curve the pUC2LTR plasmid, which contains the HIV-1 2-LTR junction. Assessment of integration by Alu-PCR was performed as previously described [57]. 2LTR circles and Alu PCR assays were performed as previously described in [58]

## Results

### R1. Identification of new partners of the LEDGF/p75 PWWP domain, by yeast two hybrid

Yeast two hybrid (Y2H) is a powerful technique developed to identify partners of a complete protein or protein domains. Y2H screens performed against LEDGF/p75 have already contributed to the characterization of cellular and viral partners of this protein [26], [34], [59]. However, these screens have always used the C-terminal part of LEDGF/p75 as bait in order to identify partners specific for its p75 form. The N-terminal part, shared between the p52 and p75 forms, contains a PWWP domain, resistant to trypsin digestion [32] and involved in the selectivity of LEDGF interaction with chromatin [38]. Recently, this domain has been shown to interact specifically to histone H3 trimethylated on lysine 36 (H3K36me3) [6], [8], [9], a property conserved with other PWWP domains [1], [4], [5], [7]. However, cellular proteins that also interact with this domain may regulate this interaction. In order to identify these proteins, we performed a Y2H screen against the LEDGF PWWP domain with its WT sequence but also with K14AK16A, W21A, I42AF43A and A51P mutated sequences. These mutations disfavor PWWP interaction with cellular chromatin [15] and their use should increase the chance to identify PWWP cellular partners. A cDNA library from human brain (Clontech, #HY4004AH, batch 0060512) was used as prey of this Y2H screen because of a large protein expression profile in this organ.

Y2H screens performed in this study revealed thirteen new and relevant PWWP cellular partners (Table S1). The **P**WWP **i**nteracting **r**egions of these proteins were called herein PIR. Functionally, seven PIRs are derived from proteins involved in DNA or RNA metabolism. RLF, TRIM28, CXXC1 and TOX4 are regulators of transcription, MCM7 is a regulator of DNA replication and NOVA1 and DICER are RNA processing proteins.

### R2. Characterization of the PIR-LEDGF interactions by protein complementation assay in mammalian cells

**Figure 1:**
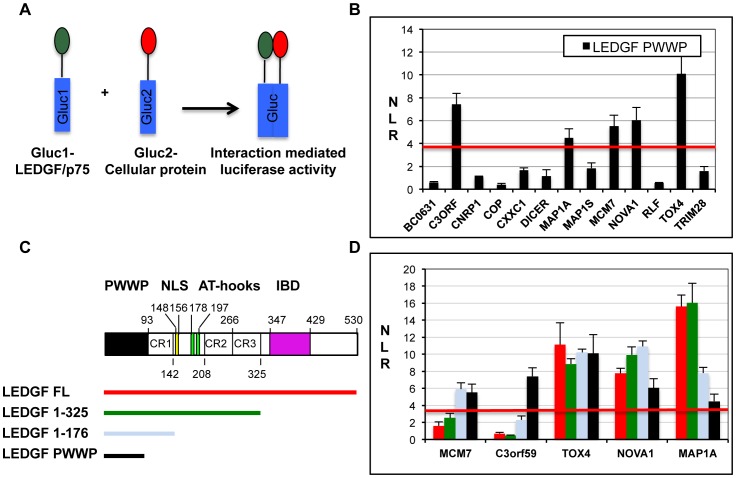
Interaction Of Pirs With Ledgf Studied By Protein Complementation Assay (Pca). A) Scheme of PCA. This assay is based on the reconstitution of the *Gaussia princeps* luciferase activity upon co-expression in 293T cell of interacting partners in fusion with two inactive fragments of the *Gaussia princeps* luciferase (Gluc1 and Gluc2). Activity of the reconstituted luciferase is measured and Normalized Luminescence Ratio (NLR) is calculated according to previous studies [50]. A threshold of NLR = 3.5 is chosen to select significant interactions. Data represent means±s.d. (error bars) from more than three independent experiments in triplicates (n>15 in B, n>5 in D). B) Interaction of 13 PIRs identified by Y2H to LEDGF PWWP measured by PCA. NLR values calculated for each PIR are represented. Five PIRs show an NLR value above the threshold corresponding to a significant interaction. C) Scheme of the LEDGF/p75 primary sequence and LEDGF constructs used for PCA study presented in D. D) Interaction of the five selected PIRs to different LEDGF constructs (FL in red, 1–325 in green, 1–176 in light blue and PWWP in black) measured by PCA. PIRs from TOX4 and NOVA1 and MAP1A show a significant interaction to the four LEDGF constructs.

To further evaluate the interaction between the LEDGF PWWP domain and the PIRs identified by Y2H, we used a **p**rotein **c**omplementation **a**ssay (PCA) based on luciferase complementation. In this split-luciferase assay, bait and prey proteins were fused to two inactive fragments of luciferase that recover their activity when brought in close proximity by interacting proteins (Figure 1A). Such PCAs using luciferase have already been used to identify and quantify protein-protein interactions in mammalian cells [50], [60]-[63]. In our study, we constructed fusions with fragments of *Gaussia princeps* luciferase (GLuc) and measured Normalized Luminescence Ratios (NLR)s corresponding to the interaction between the PWWP domain (fused to GLuc fragment 1) and the 13 PIRs (fused to the GLuc fragment 2). As reported previously, a NLR threshold of 3.5 can be used to distinguish significant interactions [50]. Consequently, among the thirteen PIRs identified by Y2H, only five PIRs were validated by this PCA with NLR values higher than 3.5 (Figure 1B). The highest and lowest values were observed for the TOX4 and MAP1A PIRs, respectively. Although the values measured for the five validated PIRs probably reflect their affinities for the PWWP domain, other parameters may influence these values. We therefore performed additional tests to validate this first set of PCA values.

The three-dimensional structure of the full-length LEDGF FL protein is still unknown [6], [40] and other parts of this protein (CRs, AT hook, IBD) may mask the PWWP domain and prevent its interaction with the PIRs. To evaluate this hypothesis, we used the PCA to measure the interaction between the five selected PIRs and three additional LEDGF constructs: the full-length (FL) sequence (1–523), the DNA and chromatin binding domains (1–325) and the PWWP domain, closest charged region and NLS (1–176) (Figure 1C). The NLRs corresponding to these interactions was compared to the NLRs already measured with the PWWP domain alone (Figure 1D). This study revealed two classes of PIRs. The first one, containing the MCM7 and C3Orf59 PIRs, is characterized by a loss of interaction with LEDGF FL. This loss is gradual for MCM7 PIR (that still interacts with LEDGF 1–176) and more severe for C3orf59 PIR (that does not interact with any other LEDGF construct). The second class of PIRs, containing TOX4, NOVA1 and MAP1A PIRs, is characterized by a conserved or increased interaction with LEDGF FL. TOX4-PIR interacts equally with the four LEDGF constructs. NOVA1-PIRs interaction with the PWWP domain is strengthened by the other parts of LEDGF N-terminal domain but this effect is inhibited by LEDGF C-terminal domain of LEDGF. Finally, MAP1A-PIR shows a preferential interaction with the two longest LEDGF constructs (FL and 1–325). Therefore, TOX4, NOVA1 and MAP1A PIRs appears to be more physiologically relevant, since their interaction with the LEDGF PWWP domain is maintained and even increased in the presence of other domains of this protein.

By PCA, we also measured the interaction between the five selected PIRs and four PWWP domains containing mutations which abolish chromatin binding (Figure S1A) [15]. Mutations chosen in this study are the same as the ones used in the Y2H screen (Table S1). The binding properties of the PIRs to the mutated PWWP domains could be divided into the same two classes as defined previously. In the first class, C3orf59 and MCM7 PIRs still interact with mutated PWWP domains (except for MCM7-PIR that doesn’t not interact with the I42A/F43A PWWP). On the contrary, the three PIRs of the second class (TOX4, NOVA1 and MAP1A), lose their interaction with the four mutated PWWP domains, with NLRs values below the positive threshold (Figure S1A). This effect is particularly important in the case of TOX4-PIR that shows a more than five fold decrease of interaction between the WT and mutated PWWP domains. These results suggest that TOX4, NOVA1 and MAP1A PIRs interact with the same surface of the PWWP domain involved in the binding of chromatin.

Finally, we tested whether the interaction observed between the five selected PIRs and the LEDGF PWWP domain could be conserved with other PWWP domains. These domains are conserved among chromatin-associated proteins and have been classified into six families [1]. In this study, we selected seven PWWP domains in addition to the PWWP of LEDGF. Two of them (HDGF and HRP2) belong to the same family as LEDGF but differ in their DNA or chromatin interaction [13], [64]. The other PWWP domains (from PDP1, MSH6, NSD2, DNMT3B and BS69 proteins) belong to different families and have already been characterized for their structure and interaction with DNA or histones [11], [12], [14], [65], [66]. These domains were tested by PCA for their interaction with the five selected PIRs (Figure S1B). PIRs belonging to the first class do not show any specificity for a given family of PWWP domains. Indeed, MCM7-PIR interacts with HDGF-related PWWP domains (LEDGF, HDGF, HRP2) but also with the PDP1 PWWP domain and C3orf59-PIR interacts with all the tested PWWP domains except that of MSH6. On the contrary, PIRs belonging to the second class (TOX4, NOVA1 and MAP1A) only interact with the PWWP domains of the HDGF family (LEDGF, HDGF, HRP2), with a strongest interaction with the HDGF PWWP domain.

In summary, three of the five selected PIRs (TOX4, NOVA1 and MAP1A) can be distinguished for their interaction with full length LEDGF protein, with the chromatin-binding surface of the LEDGF PWWP domain and with other PWWP domains of the HDGF family. These properties suggest a potential role of these proteins as regulators of the LEDGF interaction with chromatin.

### R3. Comparative localization of TOX4, NOVA1 and LEDGF proteins

**Figure 2:**
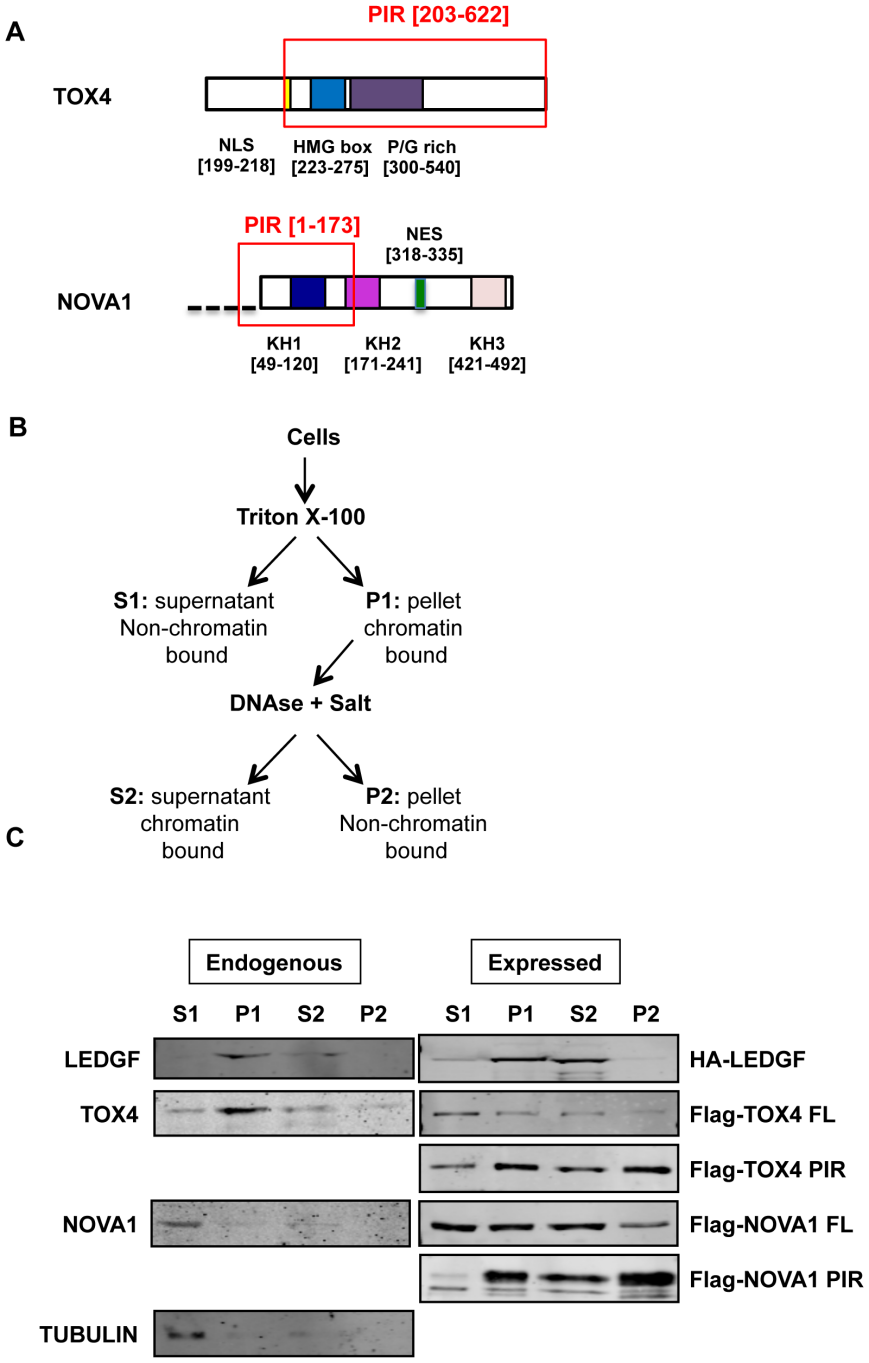
Subcellular Localization Assessed By Cellular Fractionation. A) Scheme of TOX4 and NOVA1 primary sequence with location of the PIR B) Fractionation protocol (adapted from [23]). C) Fractionation profile of Hela cells. 30µg of lysates from non-transfected cells (for expression of endogenous LEDGF, TOX4, NOVA1 and α-tubulin) or 5 µg of lysates from cells transfected with HA-LEDGF, FLAG-TOX4 PIR or FLAG-NOVA1 PIR plasmids were used in this fractionation protocol. S1 is triton-soluble fraction, P1 triton-insoluble fraction, S2, DNAse/(NH4)_2_SO_4_-soluble fraction, P2, (NH4)_2_SO_4_ fraction. Electrophoretic migrations of endogenous NOVA1 and TOX4 proteins are more precisely described in Figure S2 (lane Hela cells)

We decided to focus our study on the TOX4 and NOVA1 proteins because of their known DNA and RNA binding properties. Human TOX4 protein is a 621 aa protein, belonging to the TOX family, in which other members are known to regulate transcription of genes involved in T lymphocyte differentiation [67], [68]. The TOX4 protein contains family-conserved domains such as a N-terminal transcription activation domain (1-220), an NLS (199–218) and a HMG box involved in DNA interaction (223–275). TOX4 also possesses unique domains like a P/G rich domain (300–540) and a PNUTS binding domain located at the C-terminal part of the protein (591–621) [69] (Figure 2A). TOX4 PIR represents two thirds of the protein (203–621) and therefore lacks the transactivation domain and three aa (amino acids) of the NLS. Human NOVA1 is a neuronal splicing co-factor involved in the processing of RNAs encoding synaptic proteins [70]. The isoform 1 encodes a 510 aa protein, but other NOVA1 isoforms have been described and result from alternative splicing [71]. NOVA1 contains three conserved KH domains interacting with specific RNA sequences [72], [73] but also involved in KH dimerization [74], [75] (Figure 2A). NOVA1 also contains a NLS (24–40) and a NES (318–335) involved in the shuttling of this protein between the nucleus and cytoplasm [76]. NOVA1 PIR consists of the N-terminus of the protein (1–173) and contains the NLS and the first KH domain. Surprisingly, the PIR cloned sequence also contains 21 additional N-terminal aa coded by NOVA1 5′ untranslated region and 38 C-terminal extra aa that share no homology with published protein sequences.

We first checked the endogenous expression of TOX4 and NOVA1 proteins in different cell lines (Hela, SHSY5S and Jurkat) but also in two samples of human blood cells (activated PBMC). As shown by western blot of whole cell extracts (Figure S2), TOX4 is expressed in the different tested cells, consistent with previous results obtained with this protein [67]. We also observed an expression of NOVA1 in the different tested cells, with different isoforms probably reflecting splicing variants [71]. Previous data have shown a neuronal specific expression of this protein [77], [78] but these studies were performed using a POMA disease antisera different from the antibody used in our study (Abcam Ab97368). Moreover, other immunostaining studies have revealed the presence of NOVA1 in non-neuronal tissues or cells (http://www.proteinatlas.org/ENSG00000139910). Finally, we also observed an expression of endogenous LEDGF in the different tested cells and as expected from previously published data (data not shown).

Before studying the co-localization of these proteins, we assessed by an established cellular fractionation assay whether they were or not attached to chromatin, like the LEDGF/p75 protein [15], [23]. Briefly, this assay allows to distinguish chromatin unbound proteins (fraction S1) from chromatin bound proteins (fractions P1 and S2) and insoluble cytoskeletal and nuclear matrix proteins (fraction P2) (Figure 2B). In Hela cells, we observed a major localization of endogenous LEDGF and TOX4 in the chromatin-bound P1 and S2 fractions (Figure 2C). These two proteins are both attached to chromatin and could therefore interact between them. On the other hand, endogenous NOVA1 is mainly present in the chromatin unbound S1 fraction, although a small percentage of this protein is also present in the P1 and S2 fractions (Figure 2C). Previous studies have shown that NOVA1 is present in both cytoplasm and nucleus [76] and can colocalize in the cytoplasm with its target RNAs [76]. Therefore, NOVA1 nuclear localisation may be transient and only a small proportion of it, present in the nucleus but not tightly bound to chromatin could interact with LEDGF in the cells.

PIRs could also have a different chromatin attachement than the corresponding full-length proteins. Both PIR and full-length (FL) forms of TOX4 and NOVA1 with a N-terminal Flag epitope and HA-LEDGF were expressed in Hela cells and the same fractionation assay was applied to the transfected cells (Figure 2C). HA-LEDGF shows a clear enrichment in chromatin-bound fractions (Figure 2C). Flag-TOX4 FL is distributed between chromatin unbound and bound fractions but this partition is shifted to the chromatin bound fractions when Flag-TOX4 PIR is analysed (Figure 2C). A similar result is observed with Flag-NOVA1 FL and PIR (Figure 2C). Tubulin and LEDGF/p75 were used as internal controls for the chromatin unbound and bound fractions, respectively. Overall, these fractionation studies show that a significant proportion of TOX4 and NOVA1 (both FL and PIR) proteins is attached to chromatin and can intertact with the LEDGF FL protein.

**Figure 3:**
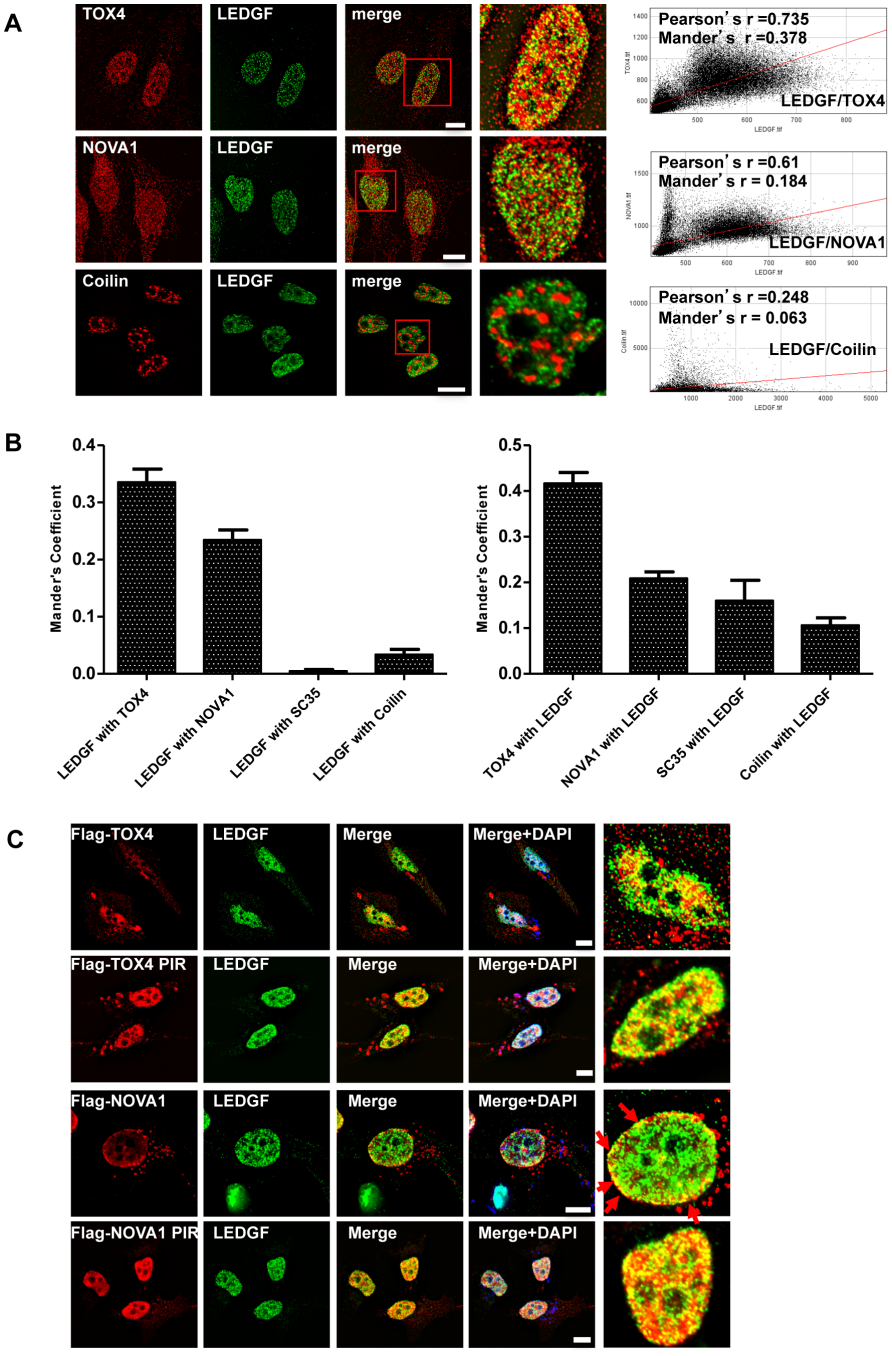
Localization Of Endogenous And Expressed Tox4 And Nova1 Proteins In Hela Cells. A) Localization of endogenous TOX4 and NOVA1 in HeLa cells. Fixed cells were co-stained with LEDGF, TOX4, NOVA1 or Coilin antibodies. Merged image is shown on right, with a zoomed panel (far right) corresponding to the red box in the merged image. The right panel corresponds to a cytofluorogram which was used to determine the degree of colocalisation using Pearsons' and Manders' coefficients. Scale bar, 10 um. B) Histogram of Mander's coefficients' for overlap of LEDGF (green) with potential partners TOX4, and NOVA1 or the non-interacting nuclear proteins Coilin and SC35 (red, left panel), or inversely, overlap of partners/Coilin/SC35 (red) with LEDGF (green, right panel). 15 individual cells were measured and error bars represent the standard error of the mean. C) Localization of expressed Flag-TOX4, Flag-NOVA1 and endogenous LEDGF in Hela cells. Fixed cells were co-stained with antibodies against Flag or LEDGF. Merged image is shown on right, with a zoomed panel of the merged image (far right). Scale bar, 10 µm.

We then studied the cellular localization of these proteins using immunofluorescence staining and epifluorescence microscopy. First, we looked at the endogenous localisation of TOX4, NOVA1 and LEDGF proteins in Hela cells. As previously described, LEDGF and TOX4 are mainly located in the nucleus and NOVA1 is present in both nucleus and cytoplasm. The amount of co-localization was quantified, and the corresponding Pearson and Mander's coefficients were calculated (plots and values for a selected cell, right panel of Figure 3A). Average Mander's coefficients were calculated for the overlap of LEDGF with TOX4, NOVA1 or non interacting controls (left panel, Figure 3B) and for the inverse overlap (right panel, Figure 3B). This study revealed a low degree of colocalisation of LEDGF with TOX4 or NOVA1 in the nucleus (Mander's coefficient between 0.2 and 0.3). However, it was significantly higher that the overlap of LEDGF with SC35, another splicing cofactor, or Coilin, another nuclear protein. Interestingly, the Mander's coefficients corresponding to the overlap of interacting proteins to LEDGF only revealed the TOX4-LEDGF co-localization as significantly higher from the others. The low degree of overlap of NOVA1 with LEDGF is probably due to the fraction present in the cytoplasm, that can not colocalize with LEDGF. Altogether, these data revealed that there is a moderate but significant colocalisation of endogenous TOX4 and NOVA1 with endogenous LEDGF/p75 which could support a functional role for the interaction of these proteins with LEDGF in the nucleus.

To further validate these observations, and to compare the localization of PIRs and full length proteins, we performed similar studies with endogenous LEDGF (detected with A300–848A antibody which detects exclusively the p75 isoform of LEDGF) and transiently expressed Flag-TOX4 and Flag-NOVA1, either full-length or PIR constructs (Figure 3C), (detected with anti-Flag M2 antibody). Using this strategy, we observed a very good co-localization of TOX4 FL and LEDGF FL that is even better between TOX4 PIR and LEDGF FL. Common foci are always present in the central part of the nucleus and exclude the nucleolus. NOVA1 and LEDGF FL also show some localization that is restricted to the inner side of the nuclear membrane (no co-localization is observed in the cytoplasm). When the NOVA1 FL construct is replaced by the PIR, this co-localization stays in the nucleus but shifts to the central part of it and becomes similar the one observed between TOX4 PIR and LEDGF. This result obtained with NOVA1 could be explained by the loss of NES in NOVA1 PIR that favors a nuclear localization and therefore the interaction with LEDGF. LEDGF could interact with NOVA1 FL during the process of nuclear export but would not cross the nuclear membrane. It is also possible that only the nuclear fraction of NOVA1 (Figure 3C) binds to LEDGF.

In addition to examining the co-localization of endogenous LEDGF with Flag-TOX4 or Flag-NOVA1 proteins (PIR or FL), HA-tagged LEDGF was co-expressed with Flag-TOX4 or Flag-NOVA1 proteins (PIR or FL) and the different proteins were localized using antibodies directed against each tag (Figure S3). Results obtained with transiently expressed HA-LEDGF are very similar to the one obtained with endogenous LEDGF. TOX4 FL co-localize with both endogenous LEDGF and HA-LEDGF and their co-localization with TOX4 PIR is more significant, especially at the nuclear periphery (Figure 3C and Figure S3, two upper panels). Similarly, NOVA1 FL co-localizes weakly with both endogenous LEDGF and HA-LEDGF and these co-localizations are increased and displaced to the inner nuclear membrane with NOVA1 PIR (Figure 3C and Figure S3, two lower panels).

In summary, co-localizations observed between TOX4 and LEDGF/p75 or NOVA1 and LEDGF/p75, either endogenous or transiently expressed proteins, support a possible interaction between them.

### R4. Interaction of TOX4 and NOVA1 (PIRs and FL) proteins to FL LEDGF protein, in cells

**Figure 4:**
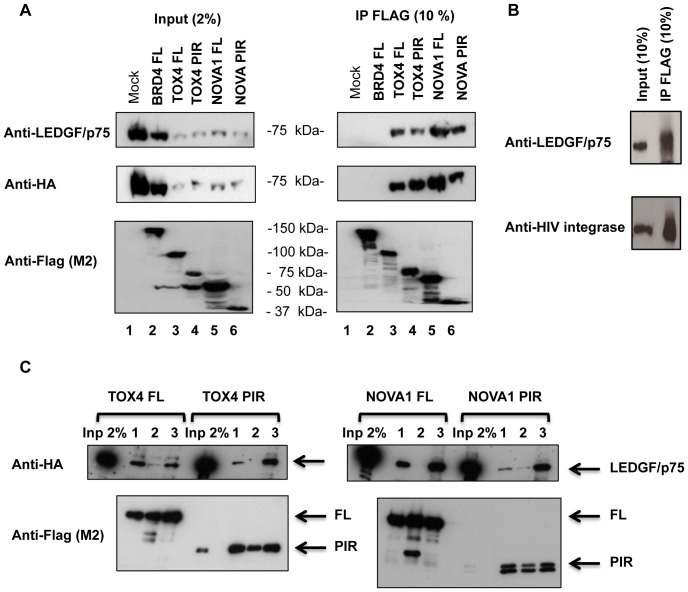
Interaction Of Tox4 And Nova1, Full Length Or Pir, With Ledgf/P75 By Co-Immunoprecipitation. Total extracts of cells transiently expressing HA-LEDGF and either 3×Flag-TOX4, 3xFlag-NOVA1, PIR or full length, Flag-HIV Integrase or Flag-Brd4 were immunoprecipitated with anti-Flag M2 coupled agarose beads. Immunoprecipitated proteins were separated on 10% or 7.5% PA-SDS gels and revealed by immunoblot using antibodies indicated on the left side of the panels and more precisely described in Material and Methods section. A) HA-tagged LEDGF co-immunoprecipitates with 3×Flag tagged full-length and PIR constructs of TOX4 and NOVA1 but not with Flag Brd4. B) HA-tagged LEDGF co-immunoprecipitates Flag-HIV1 integrase. C) DNAse (but not RNAse) treatment of cell extracts abolishes HA-tagged LEDGF co-IP with 3×Flag tagged PIR of TOX4 and NOVA1. Cell extracts were digested by nothing (lane 1), DNAse (lane 2) or RNAse (lane 3) before the co-IP protocol (IP (n>3))

Co-immunoprecipitation (co-IP) experiments were performed to confirm the interactions observed by PCA between TOX4 and NOVA1 PIRs and the full length LEDGF protein. These experiments were performed in the same cells as the PCA (293T), with a transient expression of Flag-tagged PIRs and HA-tagged LEDGF/p75 protein. This co-IP strategy was also performed with a transient expression of Flag-tagged full length TOX4 or NOVA1 proteins. Cellular extracts were immunoprecipitated with anti-Flag (M2) coupled agarose beads, separated on 10% PA-SDS gels and revealed by immunoblot using mouse anti-Flag antibody (M2) for the TOX4 and NOVA1 constructs and mouse anti-LEDGF or rat anti-HA antibodies for LEDGF (Figure 4A, lanes 3 to 6). An emply triple-Flag vector and a vector expressing Flag-integrase [52] were used as negative and positive controls, respectively, for this co-immunoprecipitation experiment (Figure 4A, lane 1 and Figure 4B). BRD4, a bromodomain binding protein that interacts with acetylated histones [79] was also used as control for the specificity of LEDGF-PIRs interaction (Figure 4A, lane 2). These co-IP experiments revealed a significant interaction between full length LEDGF and the TOX4 and NOVA1 proteins with both PIRs and full length sequences (Figure 4A). This assay confirmed the interaction between LEDGF and Flag-Integrase but no interaction was observed between BRD4 and LEDGF. This result suggests that the LEDGF-PIRs interactions observed by Co-IP do not result solely from their chromatin attachment but also require specific contact between the studied partners.

Both LEDGF, TOX4 and NOVA1 are known to interact with nucleic acids and the interaction observed by co-IP could be due to, or favored by DNA or RNA molecules present in the cellular extracts. We therefore repeated the strategy using extracts of 293T cells transtiently expressing the same proteins (Flag-tagged TOX4 or NOVA1, full length or PIR and HA-tagged LEDGF protein) and treated by DNAse or RNAse before the co-IP assay. As shown in Figure 4C, we observed a reduction in the amount of LEDGF immunoprecipitated after DNAse treatment but not after RNAse treatment, for both TOX4 and NOVA1, full length or PIR. The presence of DNA but not RNA in the extracts is therefore required for these interactions, at least under the experimental conditions of the co-IP assay.

In summary, co-immunoprecipation assays confirm the interaction observed by Y2H and PCA, between LEDGF and TOX4 or NOVA1 (full length or PIR constructs), but the presence of DNA in the extracts is necessary to observe these interactions. This result could be explained either by indirect interactions using DNA as linking molecule, or by weak and transient interactions that require a stabilization by additional partners such as nucleic acids. Further investigations were performed to test these hypothesis.

### R5. TOX4 and NOVA1 PIRS interact *in vitro* with purified LEDGF PWWP

**Figure 5:**
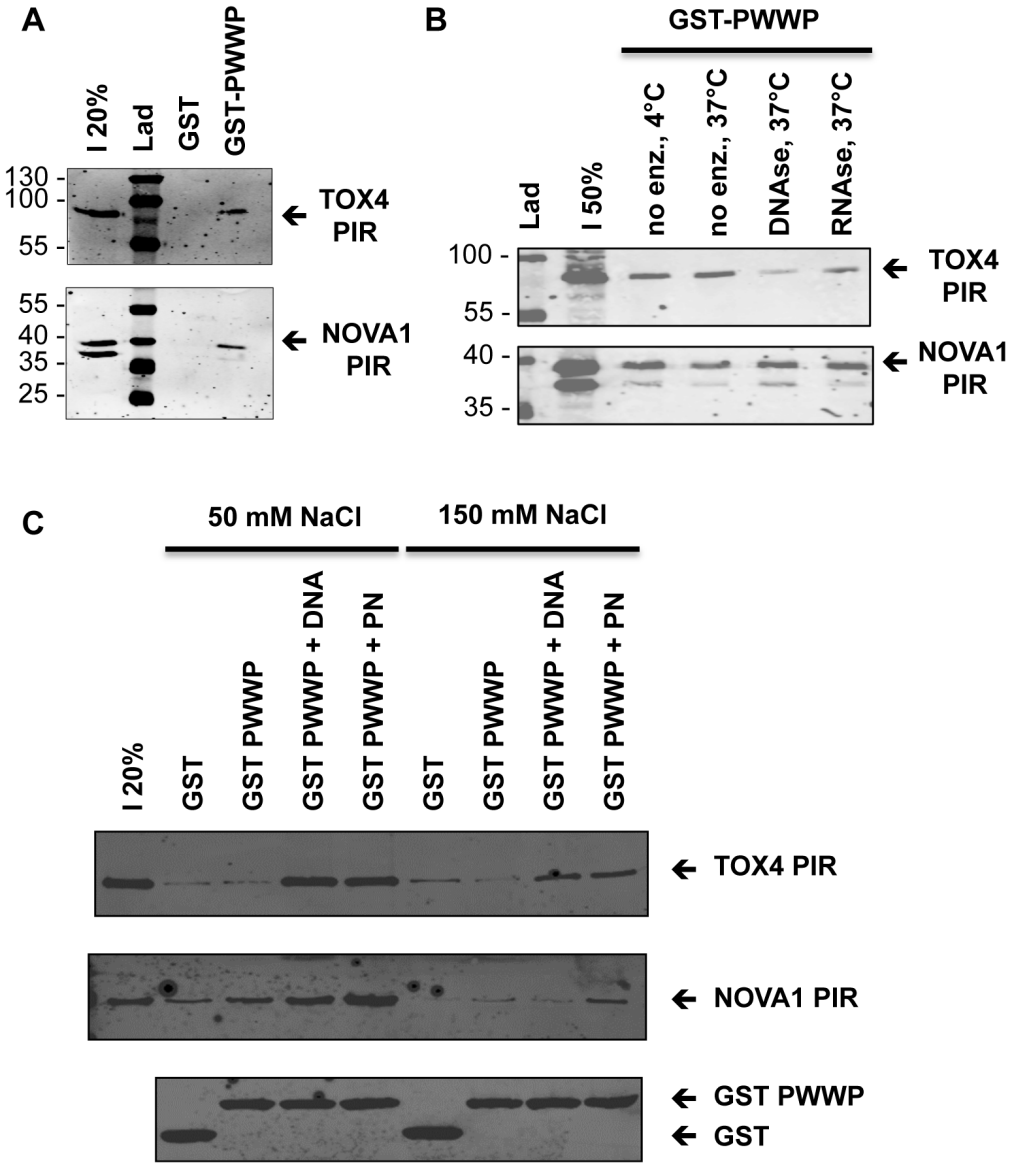
Interaction Of Tox4 And Nova1 Pirs With Ledgf Pwwp By Gst Pull-Down. GST pull down were performed using purified GST-PWWP protein and Flag-TOX4 PIR or Flag-NOVA1 PIR expressed and present in 293T cells extracts (A and B) or with His-TOX4 PIR or Flag-NOVA1 PIR expressed in *E coli* and purified (C). Eluted proteins following pull down were separated through 10% PA SDS-PAGE and analyzed by western blot using M2 anti-Flag antibody (A and B), H1029 anti-His antibody (C) and 4C10 anti-GST antibody (A to C). Purified GST was used as negative control for each experiment. B) Effect of DNA and RNA on interaction with PIRs in extracts was studied by DNAse or RNAse treatment of these extracts. C) Effect of DNA or PN on interaction with purified PIRs was studied by addition of a 2.6 kbp 5SG5E4 DNA fragment or a polynucleosome (PN) asssembled on it.

The interactions identified by PCA and co-IP, were then verified using a GST pull down approach with recombinant purified LEDGF PWWP domain. As shown on Figure 5A, GST-PWWP but not GST alone was able to pull down the TOX4 and NOVA PIRs expressed in 293T cells. The role of nucleic acids in these interactions was checked by DNAse or RNAse treatment of the extracts before their incubation to the GST-PWWP construct. As shown in Figure 5B, TOX4 binding to the PWWP was enhanced by the presence of DNA but not of RNA. Interestingly, NOVA1 binding to PWWP is not sensitive to DNAse and RNAse treatments.

To further evaluate these interactions *in vitro*, we expressed TOX4 and NOVA1 PIRs in *E Coli* with an N-terminal Histidine Tag and purified them by standard Nickel affinity purification followed by a size exclusion chromatography. Direct interaction was studied by GST pull down between the purified PIRs and GST or GST-PWWP proteins, at two salt concentrations (50 and 150 mM NaCl) (Figure 5C). Both PIRs interacted weakly with GST-PWWP, at 50 and 150 mM NaCl, but this interaction is probably non-specific since it was also observed with GST alone. When a 2.6 kbp 5SG5E4 DNA fragment (DNA) or a polynucleosome (PN) previously assembled on this fragment [49] was added during the assay, we observed a large increase of interaction of both TOX4 and NOVA1 PIRs to the GST PWWP, at 50 mM NaCl. This interaction was also observed at 150 mM NaCl for TOX4 PIR in the presence of DNA and for both PIRs in the presence of PN.

In summary, interactions of TOX4 PIR or NOVA1 PIR with LEDGF PWWP can be reproduced *in vitro* with purified proteins but they require the presence of DNA or PN. As previously concluded from co-IP assays, these interactions could either be indirect and mediated by a DNA or chromatin linking template or they are weak and require stabilizing partners such as nucleic acids or nucleoprotein complexes. Further *in vitro* studies will be required to test these hypotheses.

### R6. Effect or TOX4 and NOVA1 PIRs on HIV-1 infection

**Figure 6:**
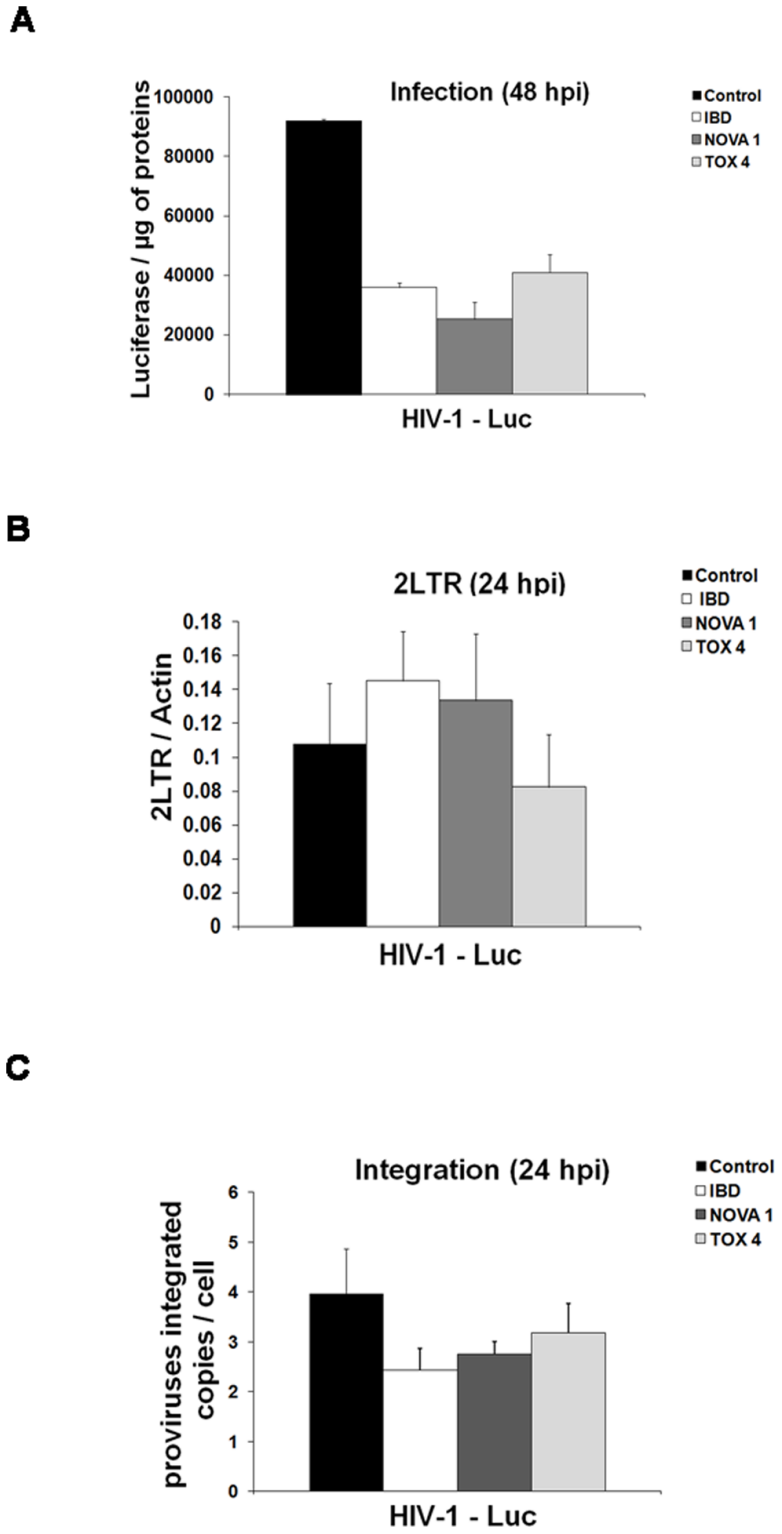
Effect On Vsv-G Pseudotyped Hiv-1 Infection Of Tox4 And Nova1 Pirs Transiently Overexpressed In Hela Cells. A) Infectivity in Hela P4 CCR5 over expressing IBD, NOVA 1 PIR and TOX 4 PIR was determined 48 hours post-infection (hpi) by measuring luciferase activity normalized to the amount of protein. B) Infections using the same virus were performed to measure the production of 2LTR circles. For this purpose, 24 hpi total genomic DNA from infected cells was used to measure 2LTR circles by real-time PCR normalized to actin. Infections carried out in the presence of Nevirapine 5 µM led to undetectable levels of both 2-LTR circles. C) Similarly, 24 hpi total genomic DNA was used to determine proviral integration sites by Alu-PCR, as described in Material and Methods. Values presented in this figure are representative of results obtained in three different experiments. Error bars correspond to one experiment performed in triplicate.

The LEDGF IBD-Integrase interaction is crucial for HIV replication and overexpression of this IBD in infected cells strongly inhibits this process by competing for the interaction with integrase [33], [35], [80]. The LEDGF PWWP domain is also important for HIV replication as it targets integrase to cellular chromatin [36], [38]. We wondered if an overexpression of the two identified PWWP partners could also affect the efficiency of replication. This question was addressed by infecting Hela CD4 CCR5 cells that transiently express Flag-TOX4 PIR, Flag-NOVA1 PIR or Flag-LEDGF IBD. The HIV-1 strain used for this study is pseudotyped for the VSV-G envelope and codes for the luciferase gene. As expected, in 3 independent experiments, we observed a significant reduction of viral infectivity in cells that transiently express the LEDGF IBD (2.2 fold effect in the experiment presented in Figure 6A). This effect is lower than the one previously observed in cells stably over-expressing GFP-IBD [33], [35]. We also observed a significant decrease of viral infectivity after a transient expression of NOVA1 or TOX4 PIRs (3.8 and 2.2 fold in the experiment presented in Figure 6A). We also tested the effect on viral replication of four other PIRs identified by the Y2H screen but not selected by PCA in 293T cells : BC0631, COP5, CNRIP1 and RLF. We observed similar levels of HIV infectivity in HeLa cells transiently expressing these proteins, while using the same conditions we detected a decrease in HIV infectivity in cells expressing the TOX4, NOVA1 or IBD constructs (Figure S4A). We also evaluated the level of expression of TOX4, NOVA1, IBD, BC0631, COP5, CNRIP1 and RLF by western blotting of the total cell extracts at the moment of virus challenge and we did not observe significant differences that could explain the effects observed on infectivity (Figure S4B).

To identify the step of HIV-1 replication targeted by these proteins, we quantified the 2LTR circles and proviruses integrated at 24 hours post infection (Figures 6B and 6C). Expression of the LEDGF IBD or the NOVA1 PIR is responsible for an integration defect, as demonstrated by an increase of 2-LTR circles and decrease of integrated copies. Expression of TOX4 PIR resulted in a slight defect in HIV integration with almost similar levels of 2LTR circles.

**Figure 7:**
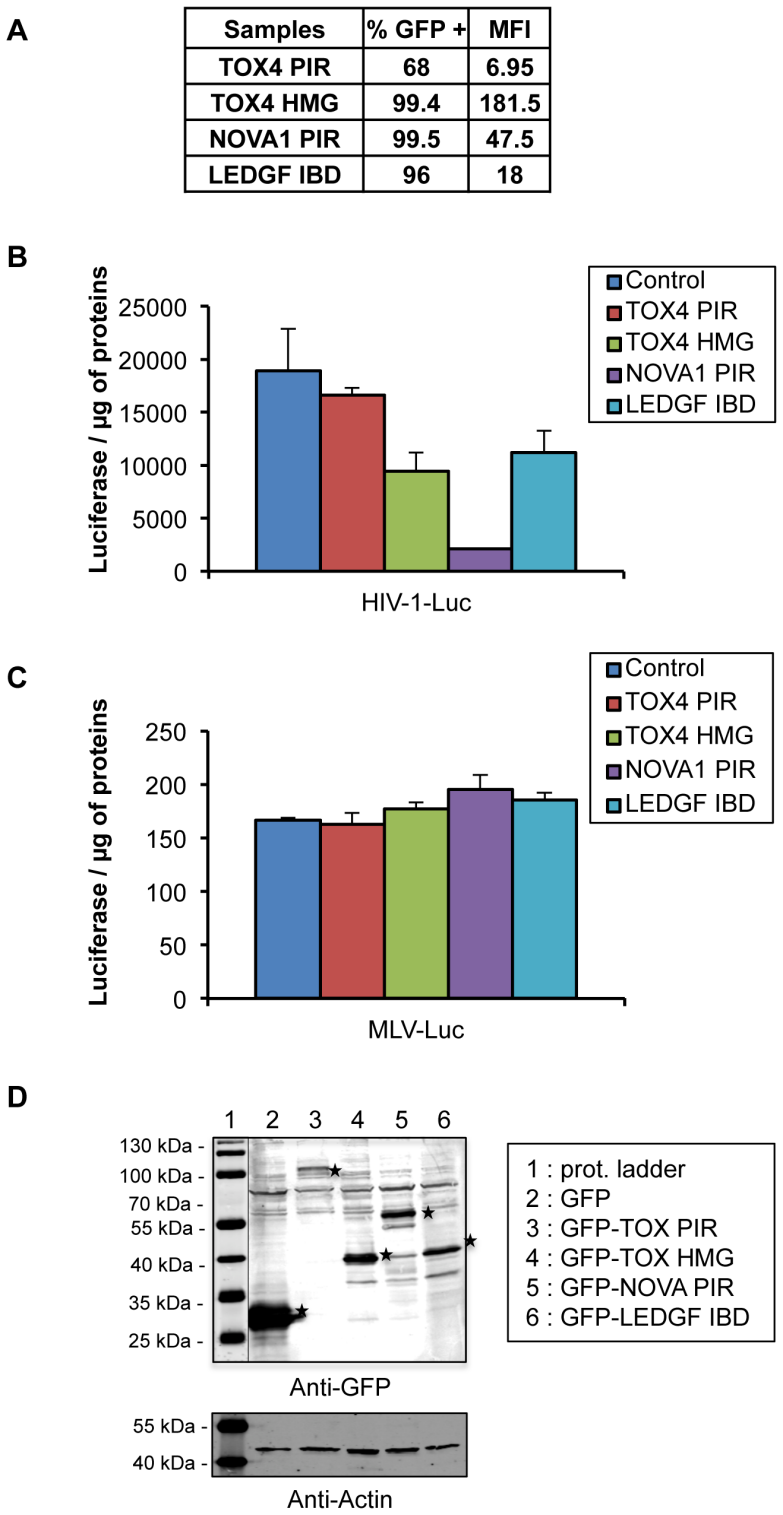
Specific Effect Of Stably Expressed Tox4 And Nova1 On Hiv-1/Vsv-G But Not On Mlv/Vsv-G Infection. A) 293T cells transduced with LVs carrying GFP-TOX4 PIR or GFP-TOX4 HMG or GFP-NOVA1 PIR or GFP- IBD (LEDGF) were analysed by FACS two weeks after transduction. 50,000 cells transduced with LVs were infected with B) 20 ng of p24 of HIV-1-Luc and C) with MLV-Luc, analyzed by luciferase assay and normalyzed by total proteins. D) Expression of EGFP-fusion proteins in infected cells. 10 µg of total cell extracts used for the infections, were separated by SDS-8% PAGE, and the presence or EGFP tagged proteins was analysed by western blotting of total extracts using an anti-GFP antibody (Abcam ab290) and normalized by actin (Abcam A5441).

Finally, if the effects of TOX4 and NOVA1 PIRs on viral replication results from an interaction between these PIRs and LEDGF/p75, they should not affect the replication of other retroviruses, like the murine leukemia virus, which integrase does not interact with LEDGF/p75. To test this hypothesis, we constructed lentiviral vectors expressing the TOX4 PIR, TOX4 HMG domain, NOVA1 PIR and LEDGF IBD sequences fused to the N-terminus of the EGFP protein and transduced 293T cells by the corresponding vectors. Bulk cells obtained after transduction were challenged with HIV-1 or MLV pseudotyped with the VSV-G envelope and coding for the luciferase as reporter gene (called HIV-1 Luc and MLV-Luc). The luciferase activity was measured after 48 hours. Surprisingly, no significant decrease of HIV infectivity was observed with the TOX4 PIR construct (Figure 7B), in contrast with a more than 2 fold decrease observed after transient expression of the same construct. This result can be attributed to a low efficiency of transduction (68% GFP positive and mean fluoresence intensity (MFI) = 6.95 in Figure 7A) resulting in a low expression of the protein in tranduced cells (Figure S4B). However, transduction with the vectors expressing the other constructs (TOX4 HMG, NOVA PIR and LEDGF IBD) was more efficient (Figure 7A) and the corresponding proteins were highly expressed in bulk transduced cells (Figure S4B). The expression of these constructs resulted in a significant decrease of HIV infectivity, the largest decrease being observed with the NOVA1 construct (Figure 7B). The decrease observed with TOX4 HMG suggested that this domain carries the PWWP interacting surface. No change of MLV infectivity was observed in cells expressing the same constructs (Figure 7C). This result supports the specificity of the role of TOX4 and NOVA1 partners of the LEDGF PWWP domain in HIV-1 infection.

## Discussion

### Two new LEDGF PWWP partners

The PWWP domain is crucial for LEDGF chromatin attachment but the molecular parameters of this interaction are still under investigation [6], [8], [23], [24], [49], [81]. This domain binds to both DNA and nucleosomes and like other PWWP domains, it also interacts with the H3K36me3 histone mark [6], [8], [9]. This interaction is probably responsible for the enrichment of LEDGF in the coding part of active genes [82]. Several nuclear events have been shown to benefit from this tethering. For example, LEDGF/p52 regulates alternative splicing and this regulation requires p52 interaction with both H3K36me3 mark and spliceosome proteins such as Srsf1 [6]. LEDGF/p75 interacts with transcription factors such as JPO2 or Menin/MLL and tethers them to chromatin [26], [28], [29]. LEDGF/p75 also interacts with lentiviral integrases and is involved in the selectivity of these enzymes for active genes in infected cells [36], [37], [39]. Altogether, these different examples of tethering highlight the role of the LEDGF PWWP-chromatin interaction. However, the bipartite interaction of the LEDGF PWWP domain to DNA and H3K36m3 histone mark [8], [9], doesn't rule out the possibility of regulation of this interaction by additional cellular cofactors.

In the present study, we identified two peptidic fragments interacting with the LEDGF PWWP domain. These two fragments, called PIRs, are derived from the TOX4 and NOVA1 protein sequences. They were obtained firstly by Y2H against the PWWP domain and their interactions with LEDGF PWWP and p75 full-length forms were confirmed in 293T cells by PCA and co-IP experiments. PCA studies revealed specific interactions of these two PIRs with the PWWP domains of the HDGF family (Figure S1B). These PWWP domains contain a PR loop which links beta sheets 2 and 3, and differs from other PWWP domains by the fact that they are able to dimerize in the presence of heparin, a molecule that mimics the negative charges of nucleic acids [83], [84]. These characteristics could be involved in TOX4 and NOVA1 PIRs interaction with the PWWP domain. We also observed that mutations of the PWWP domain selected for their effect of disrupting chromatin interaction [36], also caused the loss of interaction with the selected PIRs (Figure S1A). This result, obtained by PCA, would be consistent with results obtained by co-IP and GST pull-down experiments. There are two non exclusive interpretations for it. First, the DNA and/or chromatin binding surfaces of the PWWP domain are also used to interact with the PIRs. The consequence would be a competition between the PIRs and LEDGF interacting with DNA and chromatin and would explain the effect of PIRs overexpression on viral replication. Alternatively, both PWWP and PIRs need to bind DNA or chromatin to be found associated.

### Endogenous or transiently expressed TOX4 and NOVA1, co-localize with endogenous LEDGF

Both p52 and p75 forms of LEDGF have a ubiquitous expression and a preferential nuclear localization [25], [85]. The nuclear location and chromatin attachment are observed for LEDGF PWWP domain alone and favored by the neighbor charged region CR1 [23], [24]. We therefore investigated if the two selected PIRs or the corresponding full-length proteins co-localize with LEDGF in the cells. An initial study with endogenous TOX4 or NOVA1 revealed a weak but significant co-localization of these proteins with endogenous LEDGF (Figures 3A and 3B). The Manders's coefficients calculated for the overlaps of LEDGF with TOX4 or NOVA1 are indeed significantly higher that the ones measured for the overlaps of LEDGF with another splicing cofactor (SC35) or another nuclear protein (Coilin) (Figure 3B). Concerning TOX4 and LEDGF/p75, endogenous proteins are both clearly present in the nucleus and enriched in chromatin bound fractions (Figures 2 and 3) and their co-localization is weak but always significantly different from the co-localization of control proteins (Figures 3A and 3B). The lesser degree of colocalisation observed with NOVA1 is probably due to its dual localisation to the nucleus and cytoplasm. NOVA1 has a known role in shuttling spliced mRNA transcripts between these compartments ([76] and Figure 3). Furthermore, there are several isoforms of NOVA1 that may display different localizations and the single isoform detected by our antibody in Hela cells may be different from the several isoforms detected in PBMC cells by Western blot (Figure S2).

Interactions observed by PCA and co-IP, correspond to transiently expressed proteins. We therefore investigated if expressed TOX4 and NOVA1 proteins, in PIR or full-length form, also co-localize with endogenous LEDGF in the cells. We first observed an enrichment of the two PIRs in chromatin bound fractions with regards to the full-length proteins (Figure 2). Secondly, both PIRs significantly co-localize with endogenous LEDGF within the nucleus of the cells (Figure 3C). This co-localization is also observed with transiently expressed TOX4 FL but reduced and displaced to the inner side of nuclear membranes with NOVA1 FL (Figure 3C). This last result can be explained by a nuclear export signal present in full-length NOVA1, but absent in the PIR [76]. When the co-localization of TOX4, NOVA1 (FL or PIR) and recombinant HA-LEDGF was studied, TOX4 PIR (but not TOX4 FL) and NOVA1 (FL and PIR) all displayed a striking co-localization with LEDGF at the nuclear periphery (Figure S3A). Consistent with this, NOVA1 has previously been detected at chromosome dense regions of the inner nuclear membrane using EM [76]. Therefore, co-localization of expressed TOX4 and NOVA1 PIRs are fully consistent with interactions observed by PCA and co-IP assays. Based on these results, we decided to investigate the PIRs of these proteins in the context of HIV infection.

### Molecular models for TOX4 or NOVA1 interaction with LEDGF PWWP domain, consequences on LEDGF interaction with chromatin

Both GST pull-down and co-IP assays revealed that the LEDGF-TOX4 and LEDGF-NOVA1 interactions depend on the presence of DNA, either naked or covered by nucleosomes. Indeed, DNAse but not RNAse treatment of the cells extracts used in co-IP experiments abolished the PIR-PWWP interactions (Figure 4C) and GST pull-down assay performed with purified GST-PWWP and cell extracts pretreated with DNAse or RNAse also revealed a loss of TOX4 PIR-PWWP interaction after DNAse treatment (Figure 5B). Finally, using purified recombinant TOX4 PIR, NOVA1 PIR and GST-PWWP proteins, the studied interactions were significantly enhanced above background level (GST-alone) only in the presence of DNA or a polynucleosome template.

Are these interactions direct and biologicaly significant ? TOX4 and NOVA1 interactions to the PWWP domain were identified by Y2H and confirmed by PCA. These two approaches, although measuring activities in the nucleus, are not reported to favor the identification of nucleic acid binding proteins. Furthermore, among the several nucleic acid binding proteins identified by our Y2H screens, only three of them were positive at the first PCA test (TOX4, NOVA1 and MCM7) and the non selected nucleic acid binding proteins (RLF and CNRIP1) had no effect on HIV-1 replication (Figure S4). Among partners selected by Y2H and PCA protocols, TOX4 and NOVA1 PIRs were chosen because of their nucleic acid binding properties and their potentials to regulate LEDGF properties. The TOX4 PIR contains an HMG box that binds to DNA and damages introduced by platinium complexed molecules favor this interaction [86]. HMG boxes have also been shown to favour the DNA or nucleosome binding of transcription factors [87], [88], [89] or the remodeling of DNA structure by topoisomerase II [90]. The NOVA1 PIR contains a KH domain that binds to specific RNA sequences. We have observed an interaction of this PIR to DNA and nucleosome templates, and future investigations will clarify the affinity and specificity of these interactions. Finally, LEDGF PWWP domain binds to both DNA and nucleosomes [49] but affinities are low and both DNA and H3K36me3 mark are required for a specific and high affinity PWWP-nucleosome interaction [8], [9]. The PN used in our study is not enriched for this mark and is probably bound with a low affinity by the LEDGF PWWP domain.

In consequence, the nucleic acid binding properties of TOX4, NOVA1 and LEDGF and the required presence of DNA or PN for *in vitro* TOX4-LEDGF and NOVA1-LEDGF interactions suggest that they are not direct and that the nucleic acid or chromatin templates could serve as a bridge between the studied partners. However, we also observed that BRD4, a bromodomain protein interacting with acetylated histones [79], and used by several viral proteins as a chromatin tethering factor [91]-[93] does not interact with LEDGF in the cells. Therefore, the observed interactions between LEDGF and TOX4 or LEDGF and NOVA1 are specific and do not result solely from the DNA and chromatin binding properties of these proteins. Various interaction models, taking into account the properties of each protein can be proposed. First, both PIR-PWWP and PWWP-DNA/PN interactions could be weak and/or unstable and the presence of the three partners (PIR, PWWP, DNA or nucleosome) would be required to form stable ternary complexes. Sequential models can also be proposed, based on a conformational change induced by an initial interaction between two partners that will then favour the binding of the third partner. For example, DNA-PIRs interaction could induce a conformational change of DNA (for example its curvature) that would enhance its recognition by the PWWP domain. Alternatively, DNA binding to the PIRs or PWWP domain could induce their conformational change that would expose a new interacting surface for the other protein partner. This change could be a dimerization, like it has already been observed for the PWWP domain in the presence of Heparin [84]. These sequential models are very similar to the DNA or protein chaperon roles proposed for HMGB1 activation of p53 DNA binding [88]. However, further *in vitro* studies measuring the equilibrium and kinetic constants, with full length proteins and in the presence of DNA and chromatin templates, are required to test these different models.

What could be the consequences of TOX4 or NOVA1 interaction with the PWWP domain? Could these PIRs affect the chromatin tethering properties of LEDGF as suggested by their effect on HIV infection? The Sal3 protein interacts with the PWWP domain of DNMT3A and inhibits its CpG methylase activity [18]. Conversely, HMG boxes of HMGB1 stimulate DNA and nucleosome binding of the p53 or Rb transcription factors [87]–[89]. Further biochemical and structural investigations are required to determine if the selected PWWP-interacting partners have an inhibiting or activating effect on the LEDGF properties.

### Effect of PIRs on single round VSV-G pseudotyped HIV infection and links with the cellular roles of TOX4 and NOVA1

Overexpression of the LEDGF IBD inhibits HIV replication at the integration step, probably by competing with the LEDGF-Integrase interaction [33], [35]. Although we don't know yet if the identified PIRs stabilize or destabilize the LEDGF-chromatin interaction, we wondered if they could have an effect on HIV-1 replication, by deregulating this interaction. TOX4 and NOVA1 PIRs were selected because they bind to the PWWP domains of the two members of HDGF family interacting with lentiviral integrases. In consequence, the overexpression of these two PIRs should affect both LEDGF and HRP2 pathways of integrase activation.

Indeed, we observed a significant reduction of single round VSV-G pseudotyped HIV infection in cells that transiently express the two Flag-tagged PIRs, comparable to that obtained upon integrase IBD overexpression under our experimental conditions. Concerning NOVA1 PIR, this effect can clearly be attributed to an inhibition of the integration step. With TOX4 PIR, it is more difficult to define precisely the target step since its overexpression induces a decrease of integrated proviruses but no increase of 2-LTR viral copies with respect to control cells. A similar phenotype has been observed in HIV-infected TNPO3-depleted cells [94]. Four other PWWP partners identified by Y2H but not selected by PCA were studied for their effect on single round VSV-G pseudotyped HIV-1 infection. Their overexpression in infected cells had nearly no effect compared to overexpression of TOX4 PIR, NOVA1 PIR or LEDGF IBD (Figure S4A). This result justifies the different PCAs perfomed to select specific PWWP partners identified by Y2H.

Finally, we tested the effects of the PIRs on MLV replication since the integrase of this retrovirus does not interact with LEDGF and its replication should not be sensitive to the PIRs. Single round VSV-G pseudotyped infection by HIV-Luc and MLV luc was measured in 293T cells that stably express TOX4 PIR, TOX4 HMG, NOVA1 PIR or LEDGF IBD, fused to EGFP. Surprisingly, TOX4 PIR-expressing cells did not show any inhibition of retroviral infection. However for this construct only low efficiencies of cell transduction and protein expression were acheived. On the other hand, cells expressing TOX4 HMG, NOVA1 PIR and LEDGF IBD showed a significant decrease of single round VSV-G pseudotyped HIV infection but no effect was observed on MLV infection. This result supports the hypothesis that the viral life cycle step targeted by PIRs overexpression is LEDGF/p75-dependent.

What could be the role of TOX4 and NOVA1 during HIV-1 infection? The role of NOVA proteins and KH domains during viral replication is not very well documented. On the other hand, HMG proteins are involved in retroviral [95], [96] and non-retroviral replication [97]. HMGA protein (former HMG-I(Y)) stimulates MLV, ASV and HIV-1 integration but this activation does not require a direct interaction between integrase and the HMG protein and probably occurs through an HMG-dependent compaction of retroviral cDNA [95], [96], [98], [99]. Conversely, HMG-B1 and HMG-B2 proteins promote influenza replication by directly interacting with its nucleoprotein and enhancing its polymerase activity [97]. In both cases, the DNA binding property of the HMG protein is required for the effect on the targeted protein. Our results obtained in TOX4 HMG expressing cells suggests that this domain could be involved during HIV-1 infection.

The known functions of TOX4 and NOVA1 proteins and their link with LEDGF properties suggest different targets of these proteins during HIV-1 replication cycle.

First of all, both TOX4 and LEDGF/p75 activate transcription. TOX4 contains a strong transcription activation N-terminal domain [69]. This transcriptional activity can be repressed by protein phosphatase-1 (PP1) nuclear targeting subunit (PNUTS) that interacts with the TOX4 C-terminus. LEDGF is also a transcription co-activator of several genes involved in cellular stress response or in embryonic development [100]–[102]. The common property of these two proteins as transcription regulators could benefit each other but also proteins interacting with them. As an example, the interaction of LEDGF/p75 with lentiviral integrases regulates the selectivity of these enzymes for the elongated part of actively transcribed genes. Although this selectivity mainly results from LEDGF PWWP interaction to chromatin [37], [38], [103], [104], an additional interaction between LEDGF and the transcription machinery, mediated by TOX4 and PC4 proteins, could also be involved in this selectivity, allowing either a tracking of LEDGF by elongating polymerase complex or a recruitment of this cofactor at precise periods of the transcriptional process [105]. The biochemical characterization of nuclear complexes containing TOX4, PC4 and LEDGF, along transcribed genes will be necessary to test this hypothesis.

TOX4 and LEDGF proteins are also involved in the process of DNA repair. In fact, similar to several proteins containing an HMG box, TOX4 binds to DNA damaged by platinum anticancer drugs [86]. Complexes interacting with some of these DNA adducts also contain the LEDGF protein and proteins of the PNUTS complex. LEDGF is involved in the repair of DNA double strand breaks (DSB), by the homologous recombination pathway [106]. This process requires the C-terminal binding protein interacting protein (CtIP) that interacts with LEDGF and is tethered by this protein to specific chromatin foci through its PWWP-chromatin association. The TOX4-LEDGF interaction identified in this study could be involved in the repair of other DNA damages, like DNA adducts recognized by TOX4 HMG box or the DNA gaps generated on each side of integrated lentiviral copy. In this last case, LEDGF could be involved in DNA repair occurring just after the integration step, through its interaction with TOX4. Interestingly, other PWWP domain proteins, like Msh6 and MUM1/EXPAND, are involved in DNA repair [12], [107], [108] but the role of TOX4 and LEDGF in these repair pathways is not known.

NOVA1 regulates alternative splicing of neuronal pre-mRNAs that contain repeats of the (YCAY) binding element [70], [109]–[112]. The interaction between a chromatin binding protein and transcription regulator (LEDGF/p75) and an mRNA binding factor (NOVA1) provides a new example of link between chromatin structure, RNA Pol II transcription and mRNA processing [113], [114]. Indeed, several chromatin “readers” like the MRG15, GCN5, CHD1 and HP1 proteins interact with proteins of the splicing machinery (PTB, U2snRNP or hnRNPs) and regulate alternative splicing [114]–[117]. The mechanisms of this regulation are still under debate with complementary models favoring either the kinetics of transcription or a tethering between transcribed chromatin and synthesized RNA. It is also possible that NOVA1 interacts with LEDGF/p52 that has recently been shown to modulate splicing through its interaction with the Srf1 proteins and the H3K36m3 mark, enriched in exons [6]. The identification of NOVA1 as a LEDGF partner therefore provides a new example of co-regulation of chromatin structure/transcription/alternative splicing at specific genes where mRNA is recognized by KH domains. Comparing LEDGF chromatin loci (by Chip-seq) and NOVA1 RNA binding sites (by Hit-Clips) in the same cells would be a good start to test this possible co-regulation.

Recent studies have shown that several cellular factors, like LEDGF/p75 protein, influence the distribution of HIV-1 integration sites along the genome of infected cells [118]–[121]. Future studies will highlight whether NOVA1 and TOX4 are also involved in the distribution of HIV-1 integration sites in the host genome. A better knowledge of the interacting surfaces between the PWWP and PIRs and the identification of mutants disrupting these interactions will also help to determine the precise role of these two proteins during viral replication.

## Supporting Information

Figure S1 **Interaction Of Five Selected Pirs With Different Pwwp Domains Studied By Pca.** A) Effect of chromatin binding mutations on LEDGF PWWP domain interaction to the PIRs. NLRs corresponding to PCA performed with WT, K14AK16A, A51P, W21A or I42AF43A LEDGF PWWP domains and five selected PIRs are represented. B) Interaction of PIRs to different PWWP domains. NLR values corresponding to PCA performed with LEDGF, HRP2, HDGF, Pdp1, MSH6, NSD2, DNMT3B or BS69 PWWP domains and five selected PIRs are represented.
(TIF)

Figure S2 **Expression Of Tox4 And Nova1 In Different Cells.** Western blot of 30 µg of whole cell lysates harvested in RIPA buffer from Hela, SHSY5Y, Jurkat cell line or stimulated PMBC cells from two patients (1 and 2). Migration of TOX4 and the two predominant isoforms of NOVA1 are indicated with arrows on the left of the panel.
(TIF)

Figure S3 **Localization In Hela Cells Of Expressed Pirs (Fl Or Pir) Compared To Expressed Ledgf Fl.** Localization of TOX4 and NOVA1 (Flag-tagged, FL or PIR) and LEDGF FL (HA-tagged) in Hela cells. Scale bar, 10 µm.
(TIF)

Figure S4 **Effect Of Different Pwwp Cellular Partners Identified By Y2h On Single Round Vsv-G Pseudotyped Hiv-1 Infection.** A) Effect of several PWWP partners on HIV-1/VSV-G infection. HeLa cells were transiently transfected with FLAG-TOX4 PIR, FLAG-NOVA1 PIR, FLAG-IBD (LEDGF)PIR, FLAG-BC063142 PIR, FLAG-COP5 PIR, FLAG-CNRIP1 PIR, FLAG-RLF PIR, and infected 48 h later with HIV-1-Luc. Infectivity was determined 48 h post-infection (hpi) by measuring luciferase activity normalized to the amount of protein. B) expression of PWWP partners and LEDGF IBD constructs in Hela infected cells. 10 µg of total cell extracts were separated by SDS-10% PAGE, and the presence or Flag tagged proteins was analysed by western blotting of total extracts using anti-Flag antibody (Sigma M2). Anti Actin antibody (Sigma, A5441) was used to compare the quality of the extracts.
(TIF)

Table S1 **List Of Cellular Partners Of Ledgf Pwwp Identified By Yeast Two Hybrid (Y2h).** Ensembl Gene ID, gene name and protein description and number of Y2H hits are indicated for each partner identified.
(DOCX)
