# Supplementary figures and images for: TOX4 and NOVA1 Proteins Are Partners of the LEDGF PWWP Domain and Affect HIV-1 Replication
Source: PLoS One. 2013 Nov 27;8(11):e81217. doi: 10.1371/journal.pone.0081217 (PMC3842248; doi:10.1371/journal.pone.0081217)

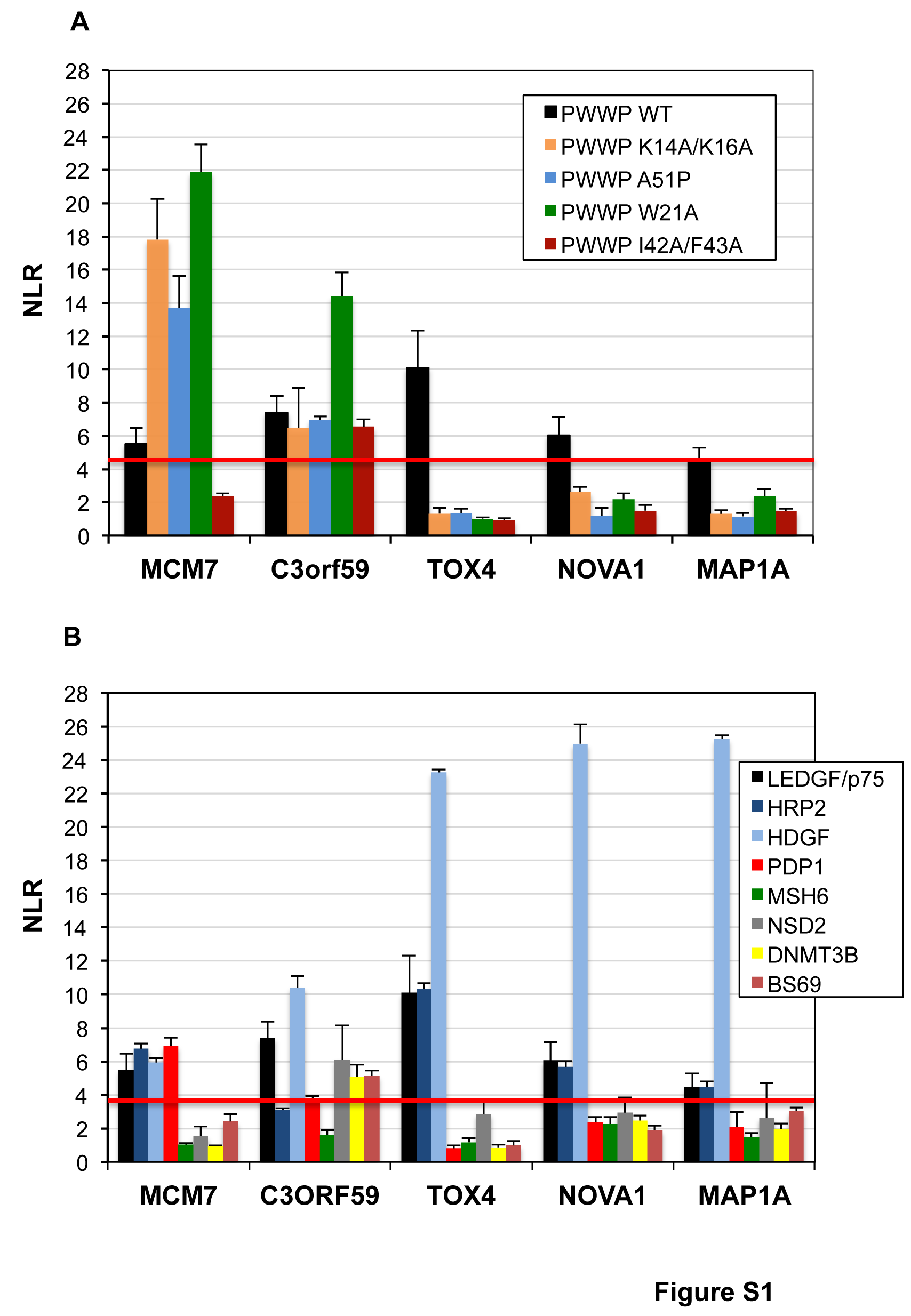

Supplement: Figure S1 — Interaction Of Five Selected Pirs With Different Pwwp Domains Studied By Pca. A) Effect of chromatin binding mutations on LEDGF PWWP domain interaction to the PIRs. NLRs corresponding to PCA performed with WT, K14AK16A, A51P, W21A or I42AF43A LEDGF PWWP domains and five selected PIRs are represented. B) Interaction of PIRs to different PWWP domains. NLR values corresponding to PCA performed with LEDGF, HRP2, HDGF, Pdp1, MSH6, NSD2, DNMT3B or BS69 PWWP domains and five selected PIRs are represented. (TIF) [file pone.0081217.s001.tif]

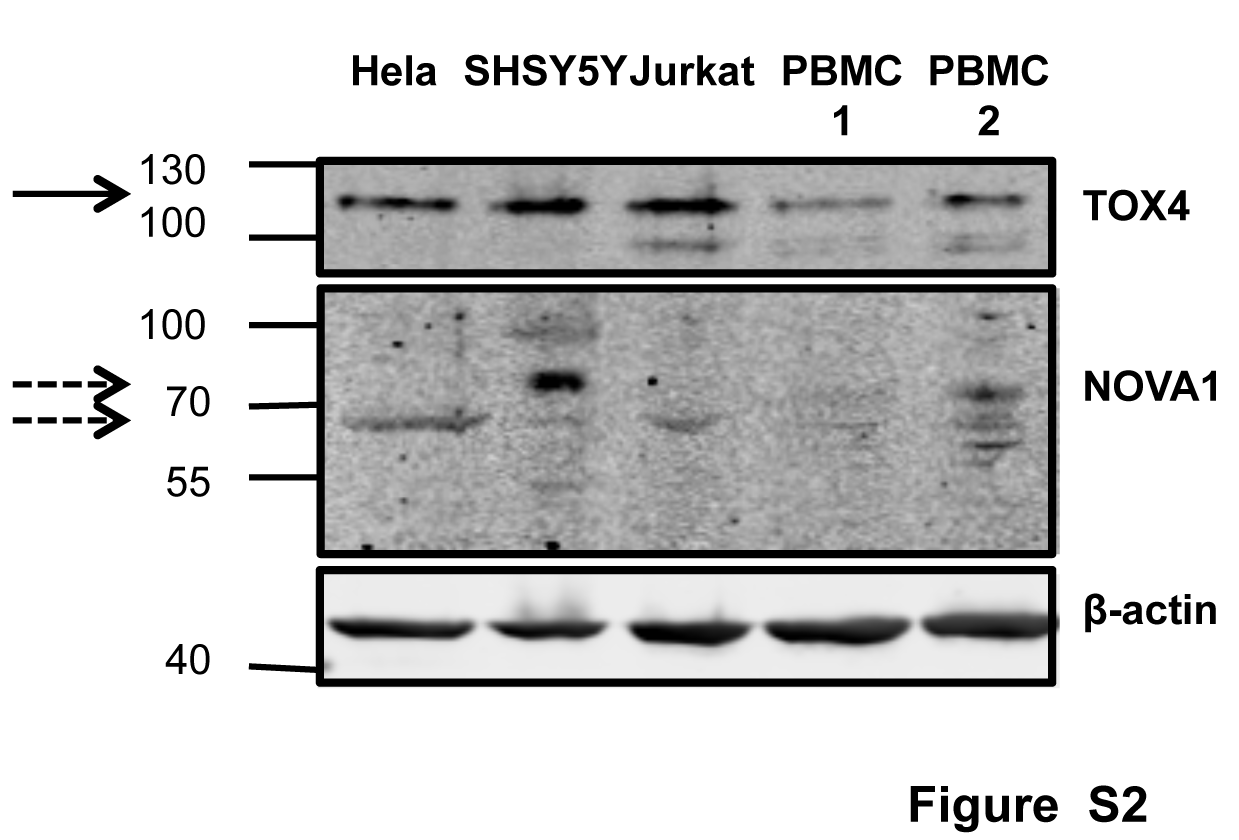

Supplement: Figure S2 — Expression Of Tox4 And Nova1 In Different Cells. Western blot of 30 µg of whole cell lysates harvested in RIPA buffer from Hela, SHSY5Y, Jurkat cell line or stimulated PMBC cells from two patients (1 and 2). Migration of TOX4 and the two predominant isoforms of NOVA1 are indicated with arrows on the left of the panel. (TIF) [file pone.0081217.s002.tif]

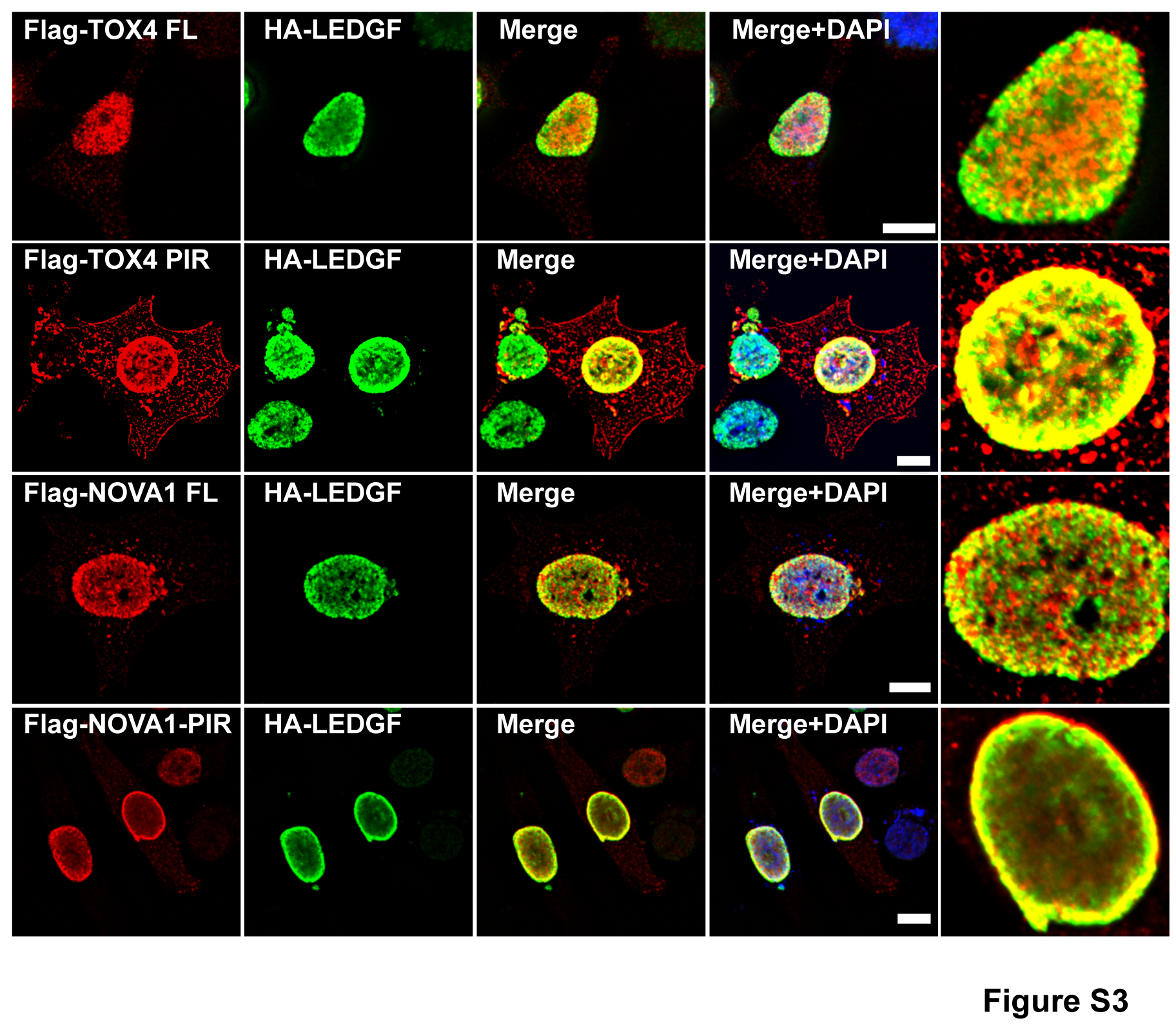

Supplement: Figure S3 — Localization In Hela Cells Of Expressed Pirs (Fl Or Pir) Compared To Expressed Ledgf Fl. Localization of TOX4 and NOVA1 (Flag-tagged, FL or PIR) and LEDGF FL (HA-tagged) in Hela cells. Scale bar, 10 µm. (TIF) [file pone.0081217.s003.tif]

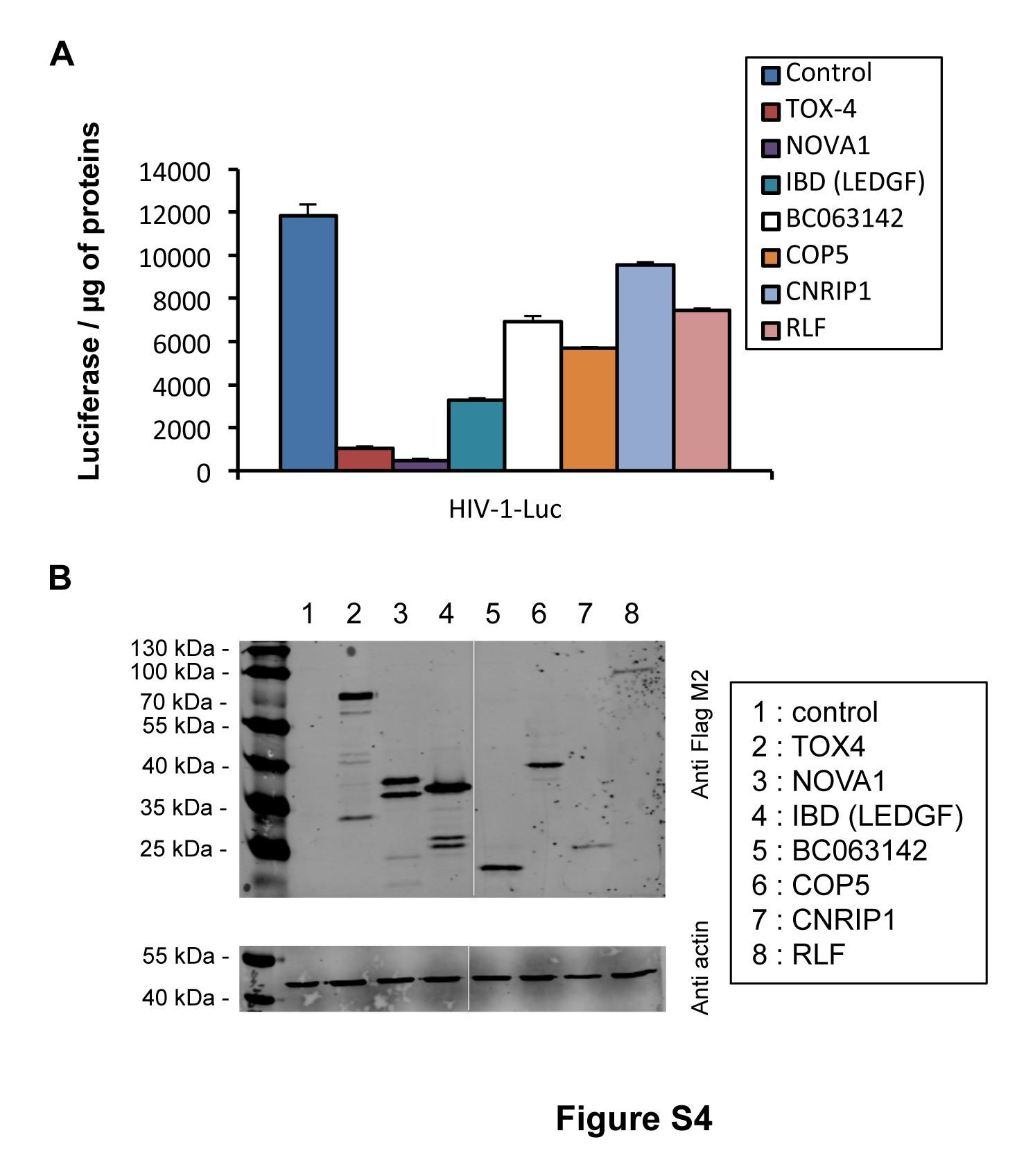

Supplement: Figure S4 — Effect Of Different Pwwp Cellular Partners Identified By Y2h On Single Round Vsv-G Pseudotyped Hiv-1 Infection. A) Effect of several PWWP partners on HIV-1/VSV-G infection. HeLa cells were transiently transfected with FLAG-TOX4 PIR, FLAG-NOVA1 PIR, FLAG-IBD (LEDGF)PIR, FLAG-BC063142 PIR, FLAG-COP5 PIR, FLAG-CNRIP1 PIR, FLAG-RLF PIR, and infected 48 h later with HIV-1-Luc. Infectivity was determined 48 h post-infection (hpi) by measuring luciferase activity normalized to the amount of protein. B) expression of PWWP partners and LEDGF IBD constructs in Hela infected cells. 10 µg of total cell extracts were separated by SDS-10% PAGE, and the presence or Flag tagged proteins was analysed by western blotting of total extracts using anti-Flag antibody (Sigma M2). Anti Actin antibody (Sigma, A5441) was used to compare the quality of the extracts. (TIF) [file pone.0081217.s004.tif]
