# Supplementary material for: TOX4 and NOVA1 Proteins Are Partners of the LEDGF PWWP Domain and Affect HIV-1 Replication
Source: PLoS One. 2013 Nov 27;8(11):e81217. doi: 10.1371/journal.pone.0081217 (PMC3842248; doi:10.1371/journal.pone.0081217)
Supplement: Table S1 — List Of Cellular Partners Of Ledgf Pwwp Identified By Yeast Two Hybrid (Y2h). Ensembl Gene ID, gene name and protein description and number of Y2H hits are indicated for each partner identified. (DOCX) [file pone.0081217.s005.docx]

| **Ensembl Gene ID** | **Gene name and Description** | **PIR** | **PWWP**  **WT** | **PWWP**  **K14A-K16A** | **PWWP**  **W21A** | **PWWP**  **I42A-F43A** | **PWWP**  **A51P** | **Total** |
| --- | --- | --- | --- | --- | --- | --- | --- | --- |
| [**ENSG00000079308**](http://www.ensembl.org/Homo_sapiens/geneview?gene=ENSG00000079308) | **BC063142** (Tensin1, Matrix-Remodelling associated protein) | A52 to C-ter |  |  |  | 1 |  | **1** |
| **ENSG00000180611** | **C3orf59** | full-length  (1-489) |  |  |  | 2 |  | **2** |
| **ENSG00000119865** | **CNRP1** (cannabinoid receptor interacting protein) | N35 to C-ter | 1 |  |  |  |  | **1** |
| **ENSG00000121022** | **COPS5** (COP9 signalosome complex subunit 5) | K43 to C-ter | 1 | 1 |  | 5 |  | **7** |
| **ENSG00000154832** | **CXXC1** (CXXC Finger protein 1) | C29 to C-ter |  |  |  | 3 |  | **3** |
| **ENSG00000100697** | **DICER** (ribonuclease involved in RNA interference) | A1508 to C-ter | 1 |  |  |  |  | **1** |
| **ENSG00000166963** | **MAP1A** (Microtubule-associated protein 1A) | R2591 to C-ter |  |  | 4 | 3 |  | **7** |
| **ENSG00000130479** | **MAP1S (**Microtubule-associated protein 1S) | D810 to C-ter |  |  | 1 |  |  | **1** |
| **ENSG00000166508** | **MCM7** (mini chromosome maintenance helicase 7) | 1 to V93 |  | 1 |  |  |  | **1** |
| **ENSG00000139910** | **NOVA1** (neuro-oncological ventral antigen 1) | (1-V174) + 21aa N-ter + 38aa C-ter |  |  |  |  | 1 | **1** |
| **ENSG00000117000** | **RLF** (rearranged L-myc fusion) | F1407 to C-ter | 2 |  |  |  |  | **2** |
| **ENSG00000092203** | **TOX4** (TOX high mobility group box family member 4) | V203 to C-ter |  |  |  |  | 2 | **2** |
| **ENSG00000130726** | **TRiM28** (tripartite motif-containing 28) | D394 to C-ter | 1 |  |  |  |  | **1** |
